# Haplotype‐resolved genome assembly of a male *Dioscorea alata* cultivar reveals the structure and evolution of young sex chromosomes

**DOI:** 10.1111/tpj.71091

**Published:** 2026-08-17

**Authors:** Komivi Dossa, Xiaojun Su, Yedomon Ange Bovys Zoclanclounon, Yao Chen, Muhammad Amjad Nawaz, Peng Ni, Ting Qiu, Gang Chen, Erick Malédon, Marie‐Claire Gravillon, Christophe Perrot, Elie Nudol, Lévy Laurent, Moussa Diouf, Petre I. Dobrev, Benjamin Karikari, Bertrand Fouks, Gemma Arnau, Pierre Mournet, Jianxin Wang, Hanâ Chaïr

**Affiliations:** ^1^ CIRAD UMR AGAP Institut 97170 Petit Bourg Guadeloupe France; ^2^ UMR AGAP Institut, Univ Montpellier, CIRAD, INRAE, Institut Agro F‐34398 Montpellier France; ^3^ Wuhan Benagen Technology Company Limited 430070 Wuhan Hubei China; ^4^ Rothamsted Research Harpenden AL5 2JQ UK; ^5^ Advanced Engineering School (Agrobiotek), National Research Tomsk State University Lenin Ave, 36 634050 Tomsk Tomsk Oblast Russia; ^6^ Laboratory for Research and Application of Supercritical Fluid Technologies in Agro‐Food Biotechnology National Research Tomsk State University Lenin Ave, 36 634050 Tomsk Tomsk Oblast Russia; ^7^ School of Computer Science and Engineering Central South University Changsha 410083 China; ^8^ Hunan Provincial Key Lab on Bioinformatics Central South University Changsha 410083 China; ^9^ Département de Mathématiques et Informatique, Faculté des Sciences et Techniques Université Cheikh Anta Diop BP 5005 Dakar‐Fann Dakar 10700 Senegal; ^10^ Institute of Experimental Botany Czech Academy of Sciences, Laboratory of hormonal regulations in plants Rozvojova 263 165 02 Prague 6 Czech Republic; ^11^ Department of Agricultural Biotechnology University for Development Studies, Nyankpala campus Tamale Ghana; ^12^ CIRAD UMR AGAP Institut F‐34398 Montpellier France

**Keywords:** dioecy, root and tuber crops, structural variants, genetic gain, genome

## Abstract

*Dioscorea alata* L., the most widely cultivated yam species, exhibits dioecy (XY system) and a strong male‐biased sex ratio. These two major constraints limit parental combinations and hinder breeding progress. To get insight into the sex chromosome structure and evolution in *D. alata*, we present a haplotype‐resolved, near‐complete genome assembly of the male cultivar “Kabusa,” spanning 958 Mb across 40 chromosomes. DalaChr6A is identified as the Y chromosome which exhibits early signs of heteromorphism, including a slight reduction in size (∼400 Kb difference) and a potential centromere shift relative to the X chromosome. Our findings also reveal a sex chromosome turnover between *D. alata* and *D. rotundata*. The sex chromosomes of *D. alata* are evolutionarily young (approximately 4.32 million years ago) and emerged after the divergence from the *D. rotundata* lineage. The sex‐determining region (SDR) is refined to approximately 7.6 Mb, representing approximately 44% of the Y chromosome. It contains several inversions and a divergence gradient was observed across these inversions. INV4 identified as the oldest pericentric inversion likely marks the early step in *D. alata* SDR evolution. Despite structural divergence, both X and Y chromosomes remain transcriptionally active. Among the 231 genes annotated in the SDR, 97 are sex‐biased and enriched in functions associated with floral organ formation and hormonal signaling pathways. This study enhances our understanding of sex chromosome evolution and sex determination in dioecious plants. It provides a gold‐standard reference genome for the *Dioscorea* genus and lays the foundation for accelerated breeding and genetic improvement in yam.

## Significance Statement

Young plant sex chromosomes remain poorly characterized because high‐quality assemblies of both sex haplotypes are scarce. Our haplotype‐resolved male genome of greater yam uncovers the architecture and evolutionary history of its XY chromosomes, providing new insights into sex chromosome evolution while enabling genomics‐assisted breeding.

## INTRODUCTION

Dioecy, the condition of separate male and female individuals within a species is present in only 5%–6% of flowering plants species (Renner, 2014). Dioecy poses specific challenges for plant breeding, particularly when sex expression is stable and strictly unisexual, such as the need for controlled pollination and limited access to one sex within breeding populations. However, it also offers unique opportunities, including the development of hybrid breeding strategies, exploitation of heterosis, and exploration of sex‐determining mechanisms (Barrett, 2013). Recent advances have provided insights into the genetic and molecular mechanisms underlying sex determination across a variety of plant species. In crops such as papaya (Liu et al., 2004), kiwifruit (Akagi et al., 2019; Akagi et al., 2023), and date palm (Torres et al., 2018), a two‐gene model of sex determination has been reported. This model involves two genetically linked genes located within a non‐recombining sex‐determining region (SDR), where one gene promotes the development of one sexual function and the other suppresses the opposite function (Akagi et al., 2019). In kiwifruit, the gene *FRIENDLY BOY* (FrBy) has been identified as a crucial male‐promoting factor, while another gene, *SHY GIRL* (SyGI), acts as a suppressor of female organ development (Akagi et al., 2018; Akagi et al., 2019). The interplay between these two genes is essential for determining the sexual phenotype of the plant. Their presence within the non‐recombining SDR prevents recombination, thus preserving their functional relationship across generations (Akagi et al., 2019; Torres et al., 2018). However, in persimmon (Akagi et al., 2020) and poplar (Müller et al., 2020), a one‐gene model appears to control sex differentiation, pointing to diverse evolutionary pathways. In many species, dioecy is leaky or labile, allowing for a degree of reproductive flexibility that can sometimes be leveraged in breeding programs (Barrett, 2013).

To date, more than 55 dioecious species genomes have been sequenced, with sex chromosomes identified in over 35 species (Garcia et al., 2023; Zhang, Pan, et al., 2022). However, in most cases, only one sex type has been sequenced, typically the homogametic sex, resulting in incomplete coverage of sex chromosomes in genomic data (Baránková et al., 2020; Garcia et al., 2023). A near‐complete or telomere‐to‐telomere (T2T) genome assembly is essential for addressing key biological questions covering genomics‐assisted breeding, the analysis of repetitive elements, genome organization and structure, evolutionary studies, gene discovery, and sex determination, among other areas of research (Garg et al., 2024; Liao et al., 2024; Liu et al., 2020; Massonnet et al., 2020). For instance, in maize, the T2T genome assembly has provided insights into the structural variations (SV) within centromeric regions that were overlooked in previous assemblies (Chen et al., 2023). This has led to a better understanding of how these variations influence traits like kernel size and yield, showcasing the potential for improving crop performance through informed breeding strategies (Chen et al., 2023). Moreover, genome assemblies of diploid organisms have collapsed the two homologous chromosomes into a single mosaic reference genome. While this approach simplifies the assembly process, it inherently obscures important genetic differences between the homologous chromosomes, which can be particularly significant in plant species with high heterozygosity (Zhang et al., 2020) and/or sexual chromosomes (Li & Durbin, 2024). This loss of allelic information limits the understanding of genetic variation and can affect downstream analyses, such as identifying SV, allele‐specific gene expression, and sex‐related genes in plants exhibiting dioecy.

Yams (*Dioscorea* spp.) are economically important tuber crops in tropical regions, with approximately 90% of global production concentrated in West Africa (Owusu Danquah et al., 2022). *Dioscorea alata*, commonly known as water yam or greater yam, is one of the most widely cultivated yam species due to its high productivity, resilience to environmental stresses, and rich nutritional profile (Neina, 2021). *D. alata* is reported to be strictly dioecious and mixed‐cytotype, with chromosome numbers of 2n = 40, 60, or 80 (Arnau et al., 2009). Its haploid genome size is estimated to be ∼454 Mb (Arnau et al., 2009). Breeding efforts to enhance the yield, disease resistance, and overall tuber quality of *D. alata* have been significantly hampered by its dioecious nature and sex ratio distortion toward male cultivars. This limits the availability of parental combinations for hybridization, hinders the development of improved cultivars, and slows genetic gains (Sugihara et al., 2024). To overcome these challenges, developing methods to control and manipulate sex phenotype in *D. alata* could facilitate breeding, thereby allowing for more effective selection and improvement of desirable traits. Recent genetic studies have identified Chromosome 6 as the sex chromosome in *D. alata*, supporting an XY (male heterogametic) sex determination system (Cormier et al., 2019; Cormier et al., 2021; Mondo et al., 2021). Despite these advances, the development of a reliable sex‐specific marker, as well as the elucidation of the molecular mechanisms and identification of key genes governing sex determination in *D. alata*, remain elusive.

The available reference genomes for greater yam are a collapsed assembly of two female (XX) individuals (Bredeson et al., 2022; Zhang et al., 2025). The lack of a high‐quality Y chromosome sequence restricts comprehensive sex chromosome analysis in this species. To gain insight into the sex chromosome structure and evolution in greater yam, we employed a combination of long‐reads and chromosome conformation sequencing technologies to assemble a haplotype‐resolved, near‐complete genome of a male (XY) individual. We also conducted in‐depth investigation of the SDR and analyzed the expression profiles of sex‐linked genes underpinning sex phenotype expression in *D. alata*. The genome revealed a high degree of similarity between X and Y chromosomes, with a 7.6‐Mb SDR representing a major pericentric inversion.

## RESULTS

### Genome assembly for the greater yam male cultivar “Kabusa”

**Figure 1:**
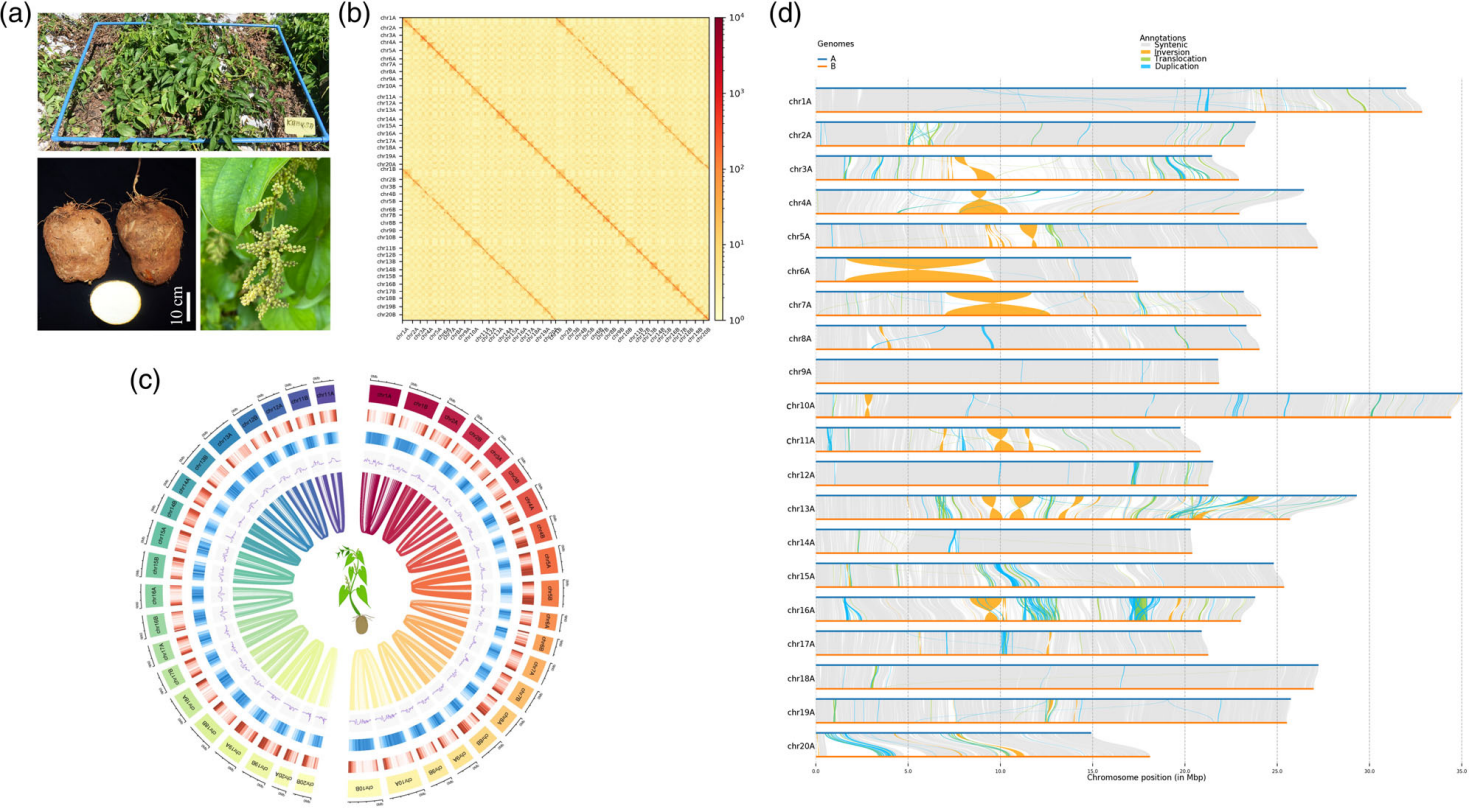
Chromosome‐level haplotype‐resolved near‐complete assembly of the male *D. alata* cultivar “Kabusa.” (a) Pictures of “Kabusa” cultivar displaying the aerial biomass in the field, the tuber shape and flesh color, and the male flowers. (b) Genome‐wide Hi‐C contact heatmap, visualized with Juicebox (Durand et al., 2016). (c) Circle diagram of basic genome information. Concentric circles from outside to inside represent chromosomes, gene density, repeat density, and GC density calculated in a 50 Kb window. Lines in the interior indicate collinearity. The scale bar = 10 cm. (d) Syntenic mapping between the two haplotypes of the “Kabusa” genome using SyRI (Goel et al., 2019). Collinear regions between HaplotypeA and HaplotypeB are indicated with gray lines, while inversion, translocation, and duplication regions are indicated with orange, green, and blue lines, respectively.

To investigate the genetic mechanisms underlying sex determination in *D. alata*, we constructed a chromosome‐level, haplotype‐resolved, near‐complete assembly of the male cultivar “Kabusa,” allowing us to generate sequences of both the X and Y sex chromosomes. “Kabusa” is the most popular cultivar in West Indies with a round‐shaped tuber, moderate resistance to anthracnose disease, and good tuber quality (Ehounou et al., 2022) (Figure 1a). The haploid genome size of “Kabusa,” a diploid cultivar (2n = 40; Figure S1), was estimated to be ∼494.13 Mb based on 19‐mers analysis, with an unsurprising high heterozygosity level (1.46%) (Tables S1, S2; Figure S2). We generated 126.5× sequence coverage of raw ultra‐long Oxford Nanopore Technology (ONT) data, 55.1× coverage of PacBio HiFi data and 123.8× coverage of chromatin conformation capture (Hi‐C) data for assembling the “Kabusa” genome (Tables S3–S5). Sequencing depths were calculated based on the estimated haploid genome size (1C).

Four assembly strategies including Hifiasm (HiFi) (Cheng et al., 2021), Hifiasm (HiFi+ONT), Hifiasm (HiFi+Hi‐C), and Nextdenovo (ONT) (Hu et al., 2024) were evaluated to identify the most effective preliminary backbone assembly to prioritize phasing accuracy. The combination of HiFi and Hi‐C reads assembled using Hifiasm produced the most robust foundation for the genome assembly with contig N50 of 12.23 Mb and 17.46 Mb for HaplotypeA and HaplotypeB, respectively (Table S6). It is worth noting that, although the Hifiasm (HiFi+ONT) assembly demonstrated good contiguity metrics, the relatively low base‐level accuracy of ONT reads may compromise phasing precision. Incorporating Hi‐C data in the assembly process theoretically enhances the accuracy of haplotype phasing results and enables chromosome scaffolding. After manual adjustment, a total of 958.71 Mb of contig sequences (contig N50 = 23.83 Mb for HaplotypeA and contig N50 = 24 Mb for HaplotypeB) were mapped to 40 chromosomes in the hybrid assembly version (Table S7; Figure 1b,c). The assembly was iteratively polished by HiFi and Illumina data. HiFi reads were aligned to the genome assembly for telomere sequence extension and repair. The telomere sequence identification results, based on the repetitive units CCCTAAA for the 5′ end and TTTAGGG for the 3′ end, are shown in Table S8 and Figure S4. Gap filling was performed using ONT and HiFi contigs as well as raw reads. After gap filling, we assessed the reliability of the filled regions by aligning both preliminary alternative assemblies and ONT reads to the gap‐filled genome. A gap was considered reliably filled if either data type provided continuous coverage spanning both flanking regions (Figure S5). In total, out of the 54 gaps detected in the assembly, 51 were successfully filled (Table S9). Only DalaChr4A, DalaChr4B, and DalaChr13B contained single gaps and 37 out of the 40 chromosomes were covered by single contigs (Table S10). A total of 99.88% of the contig sequences was anchored on the chromosomes. Altogether, statistics shown in Table 1 confirmed that we generated a highly accurate and contiguous chromosome‐level haplotype‐resolved assembly for “Kabusa.” The genome assembly is available via Yam Genome Hub (https://yam‐genome‐hub.southgreen.fr/).

**Table 1:** Summary statistics of genome assembly and gene annotation of “Kabusa”

Assembly	“Kabusa” HaplotypeA	“Kabusa” HaplotypeB	Combined
Number of chromosomes	20	20	40
Number of contigs	21	22	
Contig N50 (bp)	23 828 568	24 039 800	
Number of gaps	1	2	
Assembly length (bp)	479 656 039	480 287 338	958 714 780
Illumina read‐mapping rate (%)	99.73	99.72	
Unanchored scaffolds	5	5	10
HiFi read‐mapping rate (%)	100	100	
ONT read‐mapping rate (%)	99.65	99.62	
Genome assembly BUSCO (%)	98.1	97.9	
Genome assembly QV	53.1006	52.3324	
Annotated genes	27 767	27 609	
Genome annotation BUSCO (%)	97.5	97.8	
Mean mRNA_length (bp)	5029.52	5077.03	
Mean cds_length of per gene (bp)	1117.66	1117.43	
Mean exon_number of per gene	5.27	5.28	
Mean exon_length (bp)	302.35	300.83	
Mean intron_length (bp)	801.46	811.85	

### Annotation of the “Kabusa” genome and analysis of sequence variation between haplotypes

The annotation of the two haplotype genome assemblies revealed remarkably similar features. About 63% of each haplotype genome sequence was annotated as repetitive elements (Table S11). LTR retrotransposons were identified as the most abundant sequences (43% and 44%), of which Gypsy (20% and 21%) was the top superfamily in HaplotypeA and HaplotypeB, respectively. Moreover, 8% of each haplotype genome sequence was made of tandem repeat sequences (Table S11).

ONT‐based full‐length transcriptome data generated from eight tissues collected at developmental stages were used for genome annotation (Table S12). We employed an automated annotation pipeline supported by ONT‐based full‐length transcriptome data, *ab initio* gene prediction, and protein homology, successfully annotating 27 767 and 27 609 protein‐coding genes in HaplotypeA and HaplotypeB, respectively (Table 1; Table S13). In total, 21 652 allelic gene pairs were identified between the two haplotypes. Functional annotations were assigned to more than 94% of genes in each haplotype using multiple reference databases.

BUSCO assessment of gene annotation completeness demonstrated 97.5% and 97.8% completeness in the two haplotypes, confirming the high quality of the gene annotations. (Table S14). In addition, 139 miRNAs, 376 tRNAs, 2642 rRNAs, and 8609 snRNAs (HaplotypeA) and 140 miRNAs, 374 tRNAs, 3521 rRNAs, and 8738 snRNAs (HaplotypeB) were annotated (Table S13). We also performed putative centromere detection by resolving density of transposable elements (TE) and genes along all the 40 chromosomes, with sizes ranging from 122 139 to 5 871 010 bp (Table S15; Figures S4, S6). For example, the homologous copies of the chromosomes DalaChr6 and DalaChr20 exhibit significant differences in putative centromere position. To enhance the gene annotation of the Dala_V2 female genome assembly (Bredeson et al., 2022), we projected gene models from the high‐quality haplotype‐resolved assembly of “Kabusa” (HaplotypeA) onto the Dala_V2 assembly using Liftoff v1.6.3 (Shumate & Salzberg, 2021) with minimap2 v2.24‐r1122 (Li, 2016). A total of 27 240 genes were successfully mapped to Dala_V2, with only 527 genes failing to align, likely representing Kabusa‐specific sequences. Among the mapped genes, 99.9% were placed on chromosomal sequences. The updated annotation (Dala_V3, https://zenodo.org/records/15700131) represents an increase of 2051 genes compared to the previous version (Dala_V2, 25 189 genes), highlighting the improved completeness and accuracy of gene discovery using the “Kabusa” HaplotypeA as a reference (Table S16).

We compared the two haplotype assemblies to highlight their sequence variation. We identified 4.2 million single nucleotide polymorphisms (SNPs), including 1.17 million genic SNPs, as well as 741 026 small insertions/deletions (indels), of which approximately half were located within genic regions (Table S17). In addition, 49 694 structural variants (SVs) were found, including 5075 interchromosomal translocations, 202 inversions, 24 563 duplications, 9824 large insertions, and 10 030 large deletions (Figure 1d). Nearly half of these SVs were located in genic regions (Figure S7A). Functional enrichment analysis of SV‐influenced genes revealed a wide range of biological pathways, including nucleic acid binding, DNA integration, defense responses, post‐embryonic development, regulation of cell shape, and chloroplast organization (Figure S7B).

### Detecting the “Kabusa” Y chromosome and sex chromosome evolution in yams

The haplotype‐resolved genome of “Kabusa” offers the opportunity to determine the Y sequence that is absent in the previous female genomes (Bredeson et al., 2022; Zhang et al., 2025). *D. alata* Chromosome 6 has been identified as the sex chromosome via multiple studies (Cormier et al., 2019; Cormier et al., 2021; Mondo et al., 2021). Although discrepancies exist in the precise location of the sex‐determination region (SDR), evidence points to the presence of large SVs on the sex chromosome. The assembled DalaChr6A was slightly smaller than DalaChr6B (∼400 Kb difference) and a large pericentric inversion (from 1 619 245 to 9 202 430 bp) was found between them (Figure 1d). A total of 1087 and 1102 genes were annotated in DalaChr6A and DalaChr6B, respectively, with 840 shared genes. Aside from the single pericentric inversion, the two chromosomes exhibit strong collinearity in their distal regions, which likely correspond to the pseudoautosomal regions (PARs).

We compared the sequences of DalaChr6A and DalaChr6B to Chromosome 6 of the female genome Dala_V2 (DalaChr6F) (Bredeson et al., 2022) and found that DalaChr6A was the least collinear to DalaChr6F (Figure S8). Remarkably, the region from 1 619 245 bp to 9 202 430 bp on DalaChr6A displayed high variation with DalaChr6F. Moreover, intergenomic comparisons revealed a pericentric inversion between DalaChr6A and DalaChr6F, which was absent between DalaChr6B and DalaChr6F (Figure S9). We confirmed the existence of this inversion with HiFi long reads spanning the two breakpoints of the inverted regions (Figure S10). We therefore concluded that HaplotypeA genome in “Kabusa” contains the Y chromosome (DalaChr6A) and the pericentric inversion represents the SDR.

We aligned the “Kabusa” assemblies to the sequenced genomes of related *Dioscorea* species, focusing on chromosomes previously reported to be associated with sex determination (Figure S11). *D. alata* and *D. rotundata*, both from section Enantiophyllum (Sugihara et al., 2024), diverged approximately 17.4 million years ago (Mya) based on the synonymous substitution rate of 4 × 10^−9^ substitutions per site per year, previously estimated for Dioscorea (Hu et al., 2025). We observed strong collinearity between DalaChr6 and DrotChr6. Notably, the sex chromosome of *D. rotundata* has been identified as DrotChr11 (Tamiru et al., 2017), suggesting a sex chromosome turnover between these two species, which also differ in sex determination systems (XY vs ZW).

In more distantly related species of section Stenophora (2n = 20), such as *D. tokoro* and *D. zingiberensis*, the SDRs have not yet been fully characterized. However, previous work suggests that DtokChr3 harbors sex‐determining loci in *D. tokoro* (Natsume et al., 2022). In our analysis, part of DtokChr3 aligns with DalaChr6 (Figure S11), and a similar pattern is observed between DzinChr2 and DalaChr6. While this may reflect conservation of ancestral chromosomal segments, we cannot conclude that the SDR itself is shared. Moreover, differences in ploidy could influence genome architecture and synteny. With an increasing number of yam genome sequences becoming available (Hu et al., 2025; Ried et al., 2025), further studies are needed to identify the SDRs in these species and to determine whether the observed synteny reflects functional conservation or independent evolutionary trajectories.

### Structure of the sex‐determination region in *D. alata*

**Figure 2:**
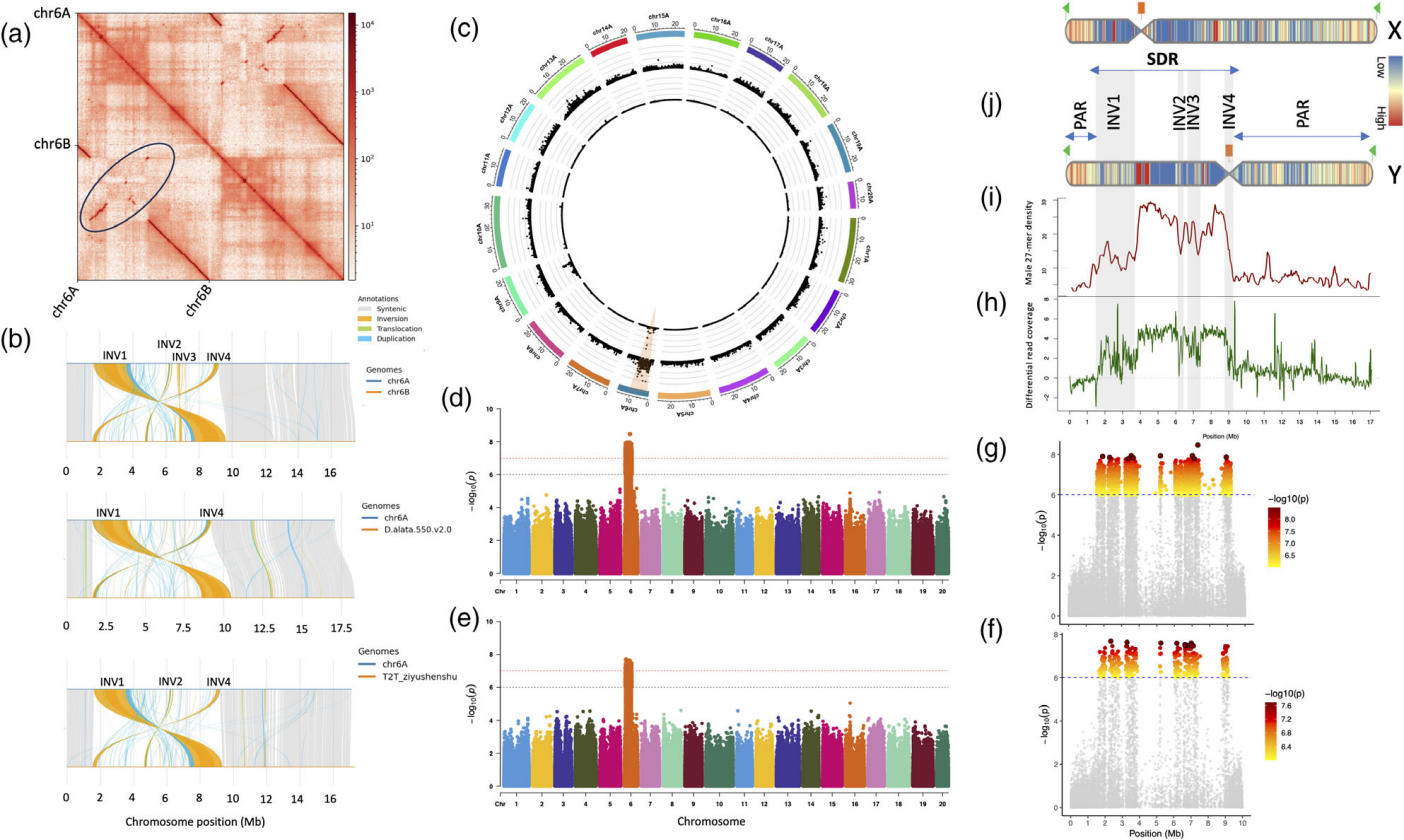
Structure of the sex determination region (SDR) in *D. alata*. (a) Hi‐C contact heatmap between DalaChr6A (Y) and DalaChr6B (X), visualized with Juicebox (Hu et al., 2024). (b) Syntenic mapping between “Kabusa” Y chromosome and X chromosomes from “Kabusa,” Dala_V2 (Bredeson et al., 2022) and Ziyushenshu (Zhang et al., 2025) using SyRI (Goel et al., 2019). Four inversions (INV1–INV4) were detected. (c) The genomic landscape of genetic variants between male and female pools. The outer track represents the 20 chromosomes (chr) of HaplotypeA in the “Kabusa” genome. The inner tracks from outside to inside represent *Fst* between females and males and male‐specific SNP density, respectively (window size 100 Kb). (d) Manhattan plot for SNP‐sex phenotype genome‐wide association study (GWAS), with the peaks indicating significant GWAS signals, and the black and red dotted horizontal lines indicating the suggestive and genome‐wide significance thresholds, respectively. (e) Manhattan plot for InDel‐sex phenotype GWAS, with the peaks indicating significant GWAS signals, and the black and red dotted horizontal lines indicating the suggestive and genome‐wide significance thresholds, respectively. Regional Manhattan plots on DalaChr6A showing the dispersion of the significant SNPs (f) and Indels (g). (h) Differential read coverage (10 Kb window) between male and female groups along the Y chromosome. (i) Exact matches of male specific 27‐mers along the Y chromosome. (j) Representation of the X (DalaChr6B) and Y (DalaChr6A) chromosomes along with the gene density (heatmap), centromere and telomere locations, pseudoautosomal regions (PAR), and the SDR containing the four inversions (INV1–INV4) detected.

To gain insights into the structure of the SDR, we generated a Hi‐C contact map for the X and Y chromosomes and further conducted a detailed comparison of the X and Y haplotypes of *D. alata* “Kabusa” using SyRI (Goel et al., 2019) (Figure 2a,b). We could observe four different inversions: INV1‐INV4 (Table 2). INV1 and INV4, located at the 5′ and 3′ boundaries, respectively, were also detected in comparisons between the Y chromosome and two independently assembled X chromosomes from female genomes (Dala_V2 (Bredeson et al., 2022) and Ziyushenshu (Zhang et al., 2025)). INV2 was also detected in the Y versus Ziyushenshu comparison, but not in Dala_V2, likely due to the lower resolution of the Dala_V2 genome. These observations support a model in which inversions have contributed to the SDR during the evolution of sex chromosomes in *D. alata*. Between the four inversions, blocks enriched in repeated sequences were observed. These regions are noncollinear with the X chromosome assemblies and may represent a putative male‐specific region of the Y chromosome (MSY), contributing to the structural differentiation of the Y chromosome. Reciprocally, the corresponding regions on the X chromosome, which lack homology with the Y, may define X‐specific regions (XSR). Interestingly, we observed that the SDR is approximately 400 Kb shorter than the homologous X region (HXR). While such size differences can occur in phased assemblies, the reduction here corresponds to specific deletions and rearrangements detected in the Y chromosome, supporting the hypothesis of a deletion event during sex chromosome differentiation (Table 2).

**Table 2:** Structure and gene content of the SDR and HXR in the “Kabusa” genome

Structure	SDR (DalaChr6A)		HXR (DalaChr6B)	
	Location/ size (Mb)	Gene content	Location: size (Mb)	Gene content
INV1	1.6–3.8/2.2	144	7.5–9.6/2.1	141
INV2	6.15–6.3/0.15	15	4.6–4.75/0.15	11
INV3	6.65–7.1/0.45	3	6.55–7/0.45	9
INV4	8.86–9.2/0.34	15	1.6–1.79: 0.19	9
MSY/XSR	−/4.46	54	−/5.11	78
Total	1.6–9.2/7.6	231	1.6–9.6/8	248

To further delineate the SDR in the *D. alata* genome, we employed five complementary analytical approaches. First, we constructed female and male read pools using resequencing data from 37 *D. alata* accessions (17 females and 20 males) from Mota et al. (2024). Reads were aligned to the HaplotypeA genome of “Kabusa” to extract variants. This yielded 15.5 million SNPs, from which we calculated the fixation index (*Fst*) between the two sex groups. The highest *Fst* values were observed between 2 and 8 Mb on DalaChr6A (Figure 2c). Second, analysis of male‐specific SNPs, defined as variants homozygous in the female pool and heterozygous in the male pool, identified 4948 such SNPs. Approximately one quarter of these variants were located between 2.6 and 9.1 Mb on DalaChr6A (Figure 2c). Third, we conducted genome‐wide association studies (GWAS) using a high‐density variant set (>2 M SNPs and 658 000 InDels) from an independent population comprising 68 males and 40 females (Figure 2d,e). Sex phenotypes were scored over 2 years at two sites in Guadeloupe (West Indies). GWAS confirmed DalaChr6 as the sole sex chromosome, with 2152 significant SNPs (1589777–9 195 813 bp) and 383 significant InDels (1638603–9 195 052 bp) associated with sex (*P* < 1e‐6; Table S18). Notably, most sex‐associated SNPs were located within the four inversions identified between Y and X (Figure 2f,g). Previously reported sex‐linked markers (Cormier et al., 2019; Cormier et al., 2021) also localized within INV1 and INV4. The colocalization of INVs with sex‐associated markers suggests that these structural rearrangements have played a central role in the evolution and differentiation of the SDR in *D. alata*. Fourth, we examined differential read coverage (10 Kb window) between male and female genotypes along the Y chromosome (Figure 2h). Several regions within the SDR displayed markedly higher coverage in males, corresponding to the boundaries of the putative MSY. These regions exhibited low variant density and contained few significant GWAS markers, suggesting suppressed recombination and high sequence divergence. Finally, we analyzed male‐specific 27‐mers (>8.2 M) along the Y chromosome, which showed a distribution pattern that precisely mirrored the male‐biased coverage (Figure 2i), further supporting the presence of Y‐specific sequences.

The SDR contains 231 annotated genes, while the HXR includes 248 genes (Table S19). Upstream, the SDR is delimited by *DalaChr6AG081680*, encoding a 3‐isopropylmalate dehydrogenase, and downstream by *DalaChr6AG083980*, encoding a transmembrane protein 50. Only 108 genes had homologous counterparts in the HXR and were considered putative gametologs. An additional five genes within the SDR showed homology with PAR‐linked genes in the X chromosome. Conversely, 118 genes within the SDR lacked identifiable X‐linked homologs and are considered Y‐specific while 140 genes are considered X‐specific. We identified 43 and 41 genes on the Y and X chromosomes, respectively, that have functional homologs on autosomal chromosomes in the *D. alata* “Kabusa” genome, including DalaChr18, DalaChr19, DalaChr2, DalaChr7, and DalaChr16A (Table S20). Notably, only four autosomal genes (from DalaChr19) have homologs located within INV1 of the SDR and HXR, while another four genes (from DalaChr16A) have homologs in the XSR. The majority of these syntenic gene pairs are found within the PARs of both chromosomes, suggesting that these relationships likely predate sex chromosome differentiation.

Together, these lines of evidence point to a region of pronounced structural and sequence divergence between the X and Y chromosomes, consistent with an evolving non‐recombining SDR (Figure 2j).

### Evolutionary divergence of the sex chromosomes in *D. alata*

**Figure 3:**
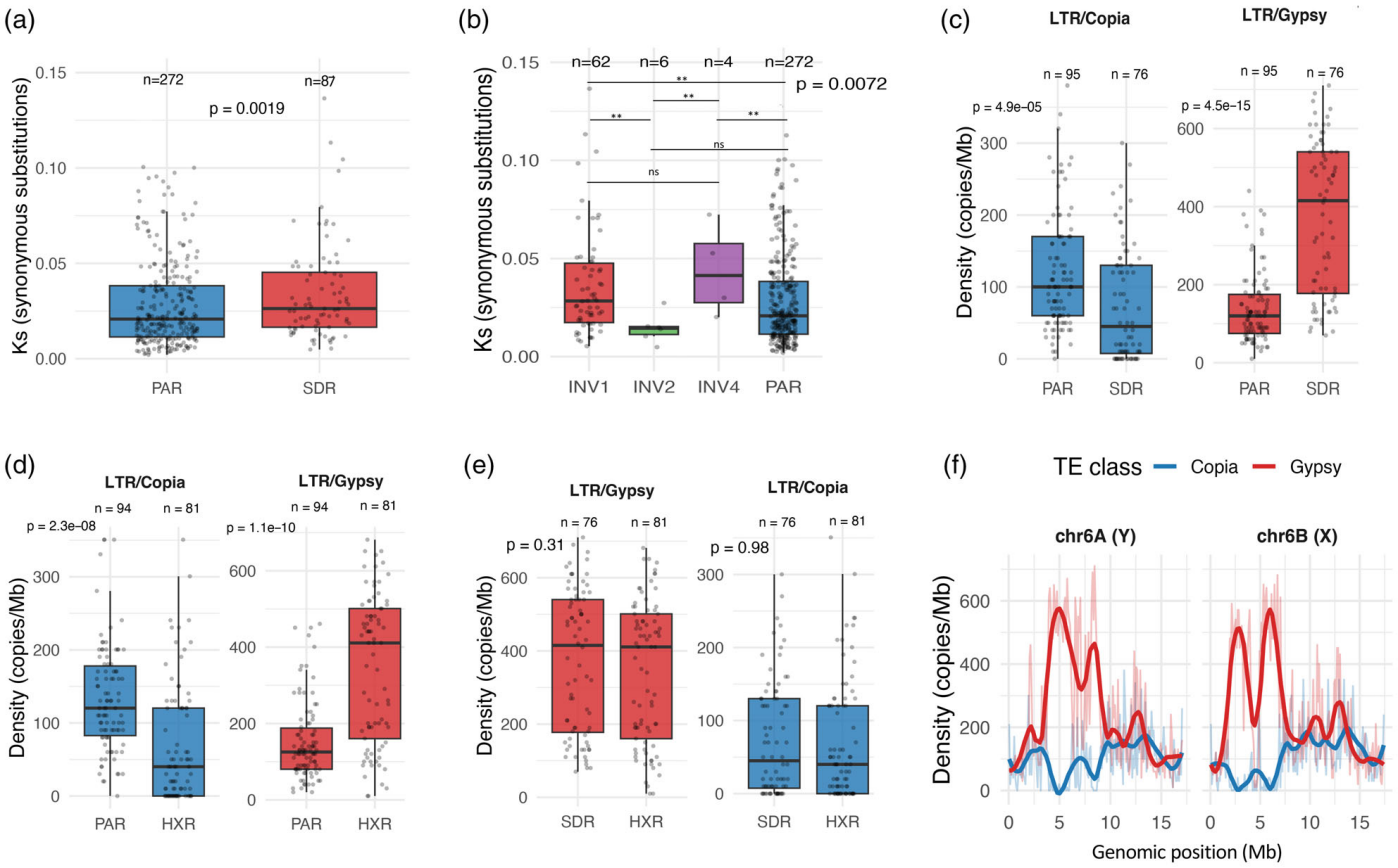
Sex chromosome divergence in *D. alata*. (a) Comparison of the synonymous substitution rates (*Ks*) of genes between SDR and PAR on the Y chromosome. (b) *Ks* comparison between Y chromosome components. ** = significant difference at *P* < 0.001. (c) Comparison of the LTR/Copia and LTR/Gypsy densities between PAR and SDR in the Y chromosome. (d) Comparison of the LTR/Copia and LTR/Gypsy densities between PAR and HXR in the X chromosome. (e) Comparison of the LTR/Copia and LTR/Gypsy densities between SDR and HXR. (f) Smoothed density plots along X and Y chromosomes. Densities are calculated per 1 Mb overlapping windows.

We computed synonymous site divergence (*Ks*) between X‐ and Y‐linked allelic gene pairs. In total, we analyzed 87 gametologs within the SDR and 272 one‐to‐one homologs genes in the PARs. After removing outliers, *Ks* values varied across the SDR–HXR segment of the sex chromosomes (range: 0.00486–0.137; mean = 0.0345; SD = 0.0259) and were significantly higher than those in the PARs (range: 0.00205–0.127; mean = 0.0292; SD = 0.0245; Kruskal–Wallis *P* = 0.0019) (Figure 3a), consistent with ongoing divergence between the SDR and the HXR. Notably, the gene *DalaChr6AG083010*, located within INV1, showed the highest *Ks* value and encodes a PLATZ transcription factor previously reported as essential for female flower morphology determination in grapevine (Iocco‐Corena et al., 2021).

To gain further insight into the evolution of the SDR, we examined *Ks* distributions within the three chromosomal inversions (INV1, INV2, and INV4) that distinguish the Kabusa Y chromosome from the three assembled X chromosomes (Figure 3b). We analyzed 74, 7, and 6 gametologs within INV1, INV2, and INV4, respectively. As expected, INV4 (range: 0.0202–0.0724; mean = 0.0438; SD = 0.0234) and INV1 (range: 0.00535–0.137; mean = 0.0367; SD = 0.0275) exhibited significantly higher *Ks* values than INV2 (range: 0.00486–0.0274; mean = 0.0145; SD = 0.00743; Kruskal–Wallis *P* = 0.0072). These results suggest that INV1 and INV4 represent ancestral chromosomal rearrangements associated with the origin of the SDR.

Using a synonymous substitution rate of *r* = 4 × 10^−9^ substitutions per site per year previously estimated for *Dioscorea* (Hu et al., 2025), we estimated that the divergence between the SDR and HXR in *D. alata* occurred approximately 4.32 Mya. The estimated divergence times of the three inversions suggest that INV4 (approximately 5.48 Mya), followed by INV1 (approximately 4.58 Mya), predate the SDR. These two inversions may therefore have played a role in the initial expansion of the SDR and the evolution of the sex chromosomes, similarly as reported in *Populus euphratica* (Zhang, Wu, et al., 2022). In contrast, INV2 exhibits a considerably more recent divergence (approximately 1.81 Mya). Altogether, these findings suggest that the sex chromosomes of *D. alata* are evolutionarily young, with their emergence occurring well after the split from the *D. rotundata* lineage (approximately 17.4 Mya). The data reveal a gradient of divergence across the inverted regions, with INV4 emerging as the oldest pericentric inversion likely involved in the early steps of SDR evolution.

TEs can influence the dynamics of the SDR by promoting recombination suppression under selective pressure, thereby shaping the organization and structural evolution of sex chromosomes (Hobza et al., 2017; Srikulnath et al., 2022). We further analyzed TE abundance and distribution pattern along chromosomes in HaplotypeA and HaplotypeB with an emphasis on LTR/Gypsy and LTR/Copia as the most abundant TEs (Figure S12). Within chromosome 6, LTR/Gypsy density was significantly higher in the SDR and HXR than in their respective flanking PARs, whereas LTR/Copia density was significantly lower in both sex‐linked regions (Figure 3c,d), indicating a localized enrichment of LTR/Gypsy in sex‐linked regions. Genome‐wide comparisons confirmed this contrasting pattern: median LTR/Gypsy density was higher in the SDR than in the haplotype A autosomal background (415 vs 170 copies/Mb) and in the HXR than in the haplotype B autosomal background (380 vs 180 copies/Mb). Conversely, median LTR/Copia density was lower in the SDR and HXR than in their respective autosomal backgrounds (40 vs 80 copies/Mb and 35 vs 90 copies/Mb, respectively). No significant difference in either LTR/Gypsy or LTR/Copia density was detected between the SDR and HXR (Figure 3e), indicating broadly similar TE compositions in the X‐ and Y‐linked regions. Smoothed density profiles along the X and Y chromosomes nevertheless revealed localized peaks of LTR/Gypsy accumulation corresponding to non‐collinear segments between the haplotypes (Figure 3f), including the putative MSY and X‐specific region. Together, these findings indicate that sex chromosome differentiation in *D. alata* is associated specifically with localized LTR/Gypsy accumulation rather than with a general increase in all LTR retrotransposon classes. Such patterns align with observations in other dioecious plant species such as papaya (Wang et al., 2012) or *Silene latifolia* (Moraga et al., 2025) where sex chromosome differentiation is associated with the accumulation/specific localization of LTR elements and chromosomal rearrangements. Although global TE density did not differ significantly between the SDR and HXR in “Kabusa,” the presence of localized LTR/Gypsy‐rich regions likely contributes to the disruption of collinearity and may play a structural role in X–Y divergence. In conclusion, our findings support a model where localized accumulation of LTR/Gypsy elements contributes to sex chromosome differentiation in *D. alata*.

### Sex‐biased gene expression along the sex chromosomes and the SDR in *D. alata*

**Figure 4:**
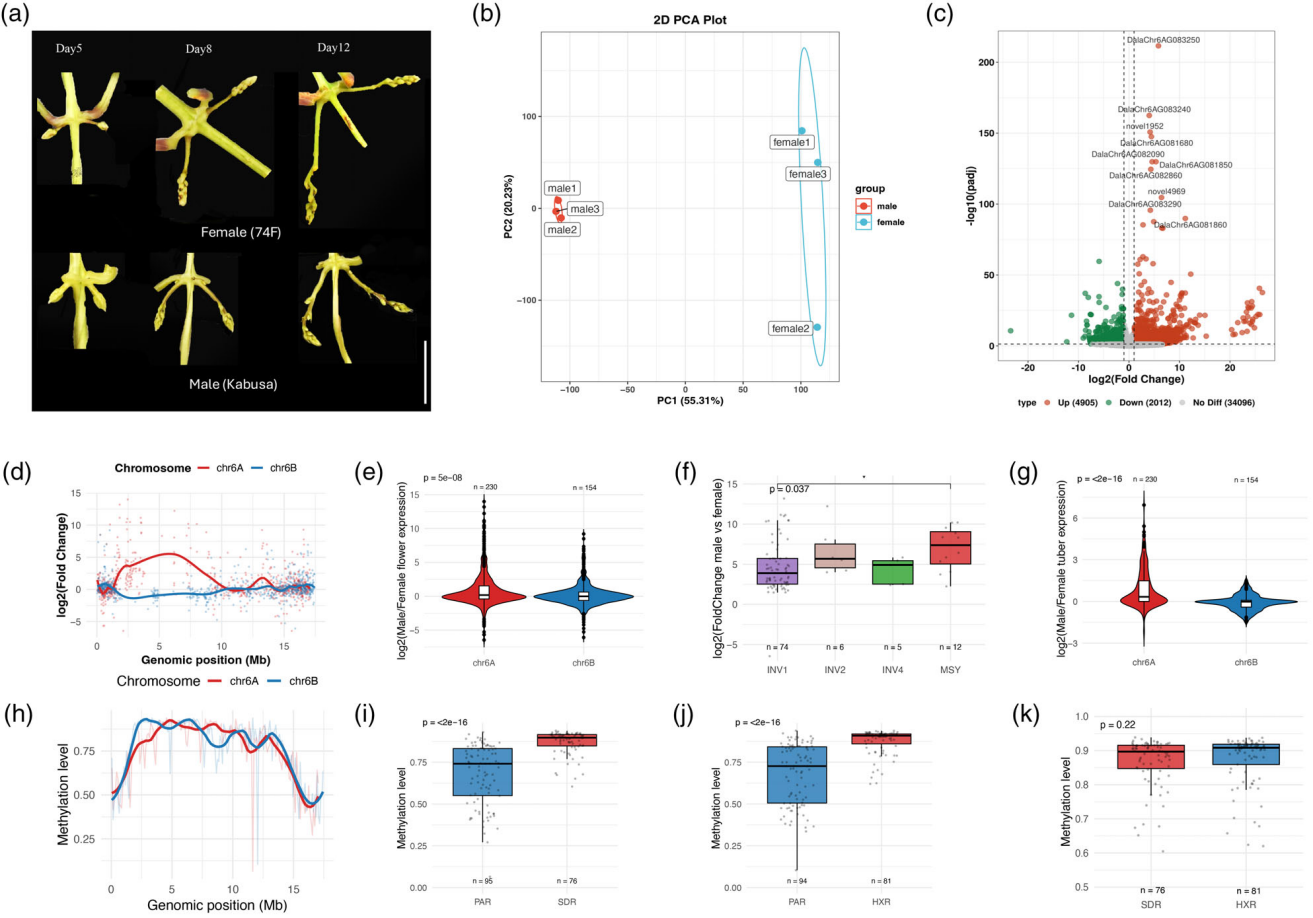
Sex‐biased gene expression in *D. alata*. (a) Development of *D. alata* flower at Day 5, Day 8, and Day 12 after blooming in male “Kabusa” and female “74F.” Ontogenetic differences between female and male flowers become morphologically evident between Day 8 and Day 12 after blooming. Scale = 1 cm. (b) Principal component analysis based on the gene expression levels for flower samples (Day 5) expressed as fragments per kilobase of transcript per million fragments mapped in the male and female groups. The first and the second principal components explaining 55% and 20% of the total variation, respectively. (c) Volcano plot displaying the differential expression analysis in flowers between male and female groups. Dots in green, red, and gray represent genes downregulated, upregulated, and not differentially expressed in male flowers compared to female flowers, respectively. (d) Gene expression patterns in flower buds (log_2_fold change between male and female groups) along the sex chromosomes. Each dot represents a sex‐biased gene (SBG). Statistical comparison of SBG located within the sex chromosomes (e) and within the Y chromosome components (f). (g) Statistical comparison of SGB in mature tuber samples located within the sex chromosomes. Samples represent mature tuber from three female *D. alata* accessions (“Dou,” “Kinabayo,” and “74F”) and three male accessions (“Divin,” “Plimbite,” and “Toufi‐Tetea”). * = significant difference at *P* < 0.05. (h) Comparison of the methylation levels (5‐methylcytosine at CpG sites) across sex chromosomes and statistical comparison between sex chromosomes components: SDR versus PAR (i), HXR versus PAR (j) and SDR versus HXR (k). Methylation levels are calculated per 100 Kb overlapping windows.

In *D. alata*, ontogenetic differences between female and male flowers become morphologically evident between Day 8 and Day 12 after blooming (Figure 4a), indicating that key sex‐determination processes likely occur during this window. To capture relevant transcriptional activity during the differentiation of sexual organs, we collected flower buds at Day 5, when a clear developmental divergence is not yet observed. Transcriptome sequencing was conducted on six female and six male individuals (Table S21). Libraries of poor quality were excluded from further analyses (Table S22). More than 918 million 150‐bp paired‐end clean reads were mapped to the haploid consensus genome (HapA + HXR) of Kabusa. While this approach enables consistent gene expression analysis, we acknowledge that some degree of cross‐mapping between homologous X and Y regions may occur, potentially introducing minor biases in quantification. These limitations are inherent to mapping in sex chromosome systems with closely related haplotypes. Transcript quantification and differential expression analysis were performed to detect the sex‐biased genes (SBG). Male and female groups were clearly separated at the transcriptional level with globally higher gene expression levels within the male group (Figure 4b; Figure S13).

Globally, male‐biased genes (4905) were twice as abundant as female‐biased genes (2012) (Figure 4c), a trend frequently reported in dioecious plant species (Prentout et al., 2020). Gene ontology enrichment analysis of the SBGs revealed several biological pathways including photosynthesis, cellular morphogenesis, pollen wall assembly, oxidoreductase activity, and carbohydrate metabolism. While not all enriched categories are directly involved in sex determination, they may reflect sex‐specific physiological processes or developmental demands during floral organ formation in *D. alata* (Figure S14). The most strongly induced SBGs were located within or in close proximity to the SDR, further supporting the central role of this region in sex differentiation (Figure 4c). A total of 751 genes on the Y chromosome (69% of total annotated Y genes) and 762 on the X chromosome (69% of total annotated X genes) were transcriptionally active in the sampled tissue types, indicating that both sex chromosomes retain substantial expression capacity and that Y chromosome of *D. alata* has not yet undergone widespread loss of gene expression typically associated with advanced stages of sex chromosome differentiation. A detailed examination of gene expression patterns along the sex chromosomes revealed a clear density of male‐biased expression along the Y chromosome (Figure 4d). In total, 230 SBGs were identified on the Y chromosome, compared to 154 SBGs on the X chromosome. As illustrated in Figure 4e, SBG located in the Y chromosome exhibited significantly higher log_2_ fold changes in expression between male and female groups, compared to their counterparts on the X chromosome (*P* = 4.994e^−08^). Along the sex chromosomes, the magnitude of differential expression was more pronounced within the SDR and HXR, highlighting a potential regulatory role of these regions in sex‐specific transcriptional activity (Figure 4d). Within the SDR, 97 SBGs were detected (Table S23), with all but one—*DalaChr6AG081990*, a *GERMIN‐LIKE PROTEIN SUBFAMILY 3*, showing higher expression in male flowers. This supports the idea that the SDR is a transcriptional hub for male‐specific functions. Notably, genes located in the MSY displayed markedly stronger expression in males, as would be expected for loci involved in male reproductive development and function (Figure 4f). In contrast, the HXR contained 45 SBGs, but only a single gene *DalaChr6BG081880*, annotated as *Cytochrome P450* was upregulated in male samples (Table S23). Interestingly, these X‐ and Y‐ linked SBGs identified in floral tissues also exhibited differential expression in mature tubers (three males *D. alata* accessions compared to three female *D. alata* accessions), suggesting that sex‐associated transcriptional divergence may extend beyond reproductive tissues in *D. alata* (Cossard et al., 2019; Zhang, Wu, et al., 2022) (Figure 4g).

Notably, we identified 10 genes within the SDR that were only expressed in males (Table S23). These genes may contribute to sex differentiation by promoting male organ development and/or repressing female organ development. For instance, *DalaChr6AG082390*, annotated as a *style cell‐cycle inhibitor 1‐A* (*SCI1*) homolog, showed strong expression in all male flower samples but was undetectable in female flowers. In *Nicotiana tabacum* and *Arabidopsis thaliana*, *SCI1* has been shown to control cell proliferation in pistil development (DePaoli et al., 2011) (DePaoli et al., 2014). While its function in male reproductive tissues has not been reported in these model species, the male‐specific expression pattern in *D. alata* raises the possibility of a lineage‐specific functional divergence or a novel regulatory role in male floral development. However, functional validation is required to confirm its role in sex differentiation in yam. Several other SBGs within the SDR have known functions in flower sexual organ development, including *DalaChr6AG081680* (*3‐isopropylmalate dehydrogenase*) essential for pollen development and proper embryo sac development (He et al., 2011); *DalaChr6AG082570* (SWR1) involved in female germline development (Huang et al., 2023); *DalaChr6AG083470* (*NYFC*) known for its role in flowering and organ morphogenesis (Siriwardana et al., 2016); and *DalaChr6AG082810* (*ARR*) involved in cytokinin (CK) hormone signaling (Luo et al., 2020). Since INV4 is the most ancestral inversion associated with sex chromosome evolution in *D. alata*, we hypothesize that SBGs located within this region may represent strong candidates for primary sex‐determining factors. Among them, four have homologs in model species with functions related to reproductive development. *DalaChr6AG083890* encodes a rhomboid‐like protein, homologous to *Arabidopsis thaliana* genes involved in pollen development and pollen wall formation (Kanaoka et al., 2022). *DalaChr6AG083930* encodes a PHD‐finger protein with strong similarity to genes essential for male fertility in *A. thaliana*, soybean, and rice (Thu et al., 2019; Yang et al., 2003; Yang et al., 2019). *DalaChr6AG083880* is a homolog of *CORONATINE INSENSITIVE 1* (*COI1*), an F‐box protein known to play a central role in jasmonic acid signaling and reproductive organ development across multiple plant species (Wasternack, 2020; Xie et al., 1998). Finally, *DalaChr6AG083840* is similar to the *MATERNAL EFFECT EMBRYO ARREST 9* gene in *A. thaliana*, which is involved in female gametophyte development and function (Pagnussat et al., 2005). While functional validation remains to be conducted in *D. alata*, the presence of these homologs highlights candidate genes potentially involved in sex determination and reproductive processes in this species.

To explore whether epigenetic regulation may contribute to the observed biased gene expression, we analyzed DNA methylation levels of 5‐methylcytosine (5mC) at CpG sites. Globally, quite similar patterns of methylation levels and distribution across homologous chromosomes of HaplotypeA and HaplotypeB could be observed (Figure S15; Figure 4h). However, a finer‐scale comparison of X and Y chromosomes revealed a significantly higher methylation level within the SDR and HXR compared to the PARs (Figure 4i,j). No significant difference was observed between the SDR and HXR, suggesting that the epigenetic landscape of this region is largely conserved despite major structural rearrangements (Figure 4k). It is important to note that our analysis focused solely on CpG methylation and did not account for other types of DNA methylation (e.g., CHG or CHH contexts), which may also play a role in gene regulation (Fernandes et al., 2024).

### Development of a reliable sex‐prediction molecular marker

A sex‐prediction PCR allele competitive extension (PACE) marker for *D. alata* was previously developed (Cormier et al., 2021). However, it does not achieve 100% accuracy in distinguishing male and female individuals. This suggests that the marker is not tightly linked to the sex determination loci on DalaChr6. In this study, we developed a new PACE marker, *sda6*, based on allelic variation within the coding sequence of one candidate sex‐controlling gene *DalaChr6AG083080* (psbP domain‐containing protein 6) and its homolog *DalaChr6BG082060*. The sda6 marker is located within the INV1. From the transcriptome data, all female genotypes were homozygous T/T (DalaChr6B: 7643820 bp) while all male genotypes were heterozygous G/T (DalaChr6A: 3714267 bp) (Table S24). Genotyping 146 *D. alata* accessions using the *sda6* marker revealed two distinct clusters corresponding to male and female groups (Figure S16). Among the 74 accessions with known sex phenotypes, *sda6* accurately distinguished male and female plants with 100% accuracy (Table S25). This result underscores *sda6* as a reliable molecular marker for predicting the sex phenotype in *D. alata*, addressing an essential requirement for advancing breeding efforts (Mondo et al., 2021).

## DISCUSSION

The haplotype‐resolved genome assembly of the “Kabusa” cultivar surpasses the quality of existing *D. alata* assemblies (Bredeson et al., 2022; Zhang et al., 2025), establishing a new reference standard for the *Dioscorea* genus. It features a significantly higher contig N50, improved genome annotation, and greater accuracy. The assembly is nearly gap‐free and haplotype‐resolved, containing a large repertoire of novel genes. Importantly, it includes the previously missing full Y chromosome sequence. As demonstrated in other plant species, such as pear (Li et al., 2024) and walnut (Han et al., 2024), haplotype‐resolved genome assemblies offer significant advantages over mosaic genomes. These include enhanced resolution for studying complex traits and evolutionary dynamics, especially in species with high heterozygosity or sex‐specific chromosomes, such as *D. alata*. The haplotype assemblies of the “Kabusa” genome provide valuable resources for a wide range of applications, including variant calling, gene expression analysis, genome‐wide association studies, genomic prediction, genome editing, and comparative genomics. Additionally, they enable precise evaluation of the specific contributions of each haplotype to phenotypic traits, supporting both functional and applied research efforts (Liao et al., 2024; Raiyemo et al., 2024).

The identification of telomeric and centromeric sequences is crucial for ensuring a complete, high‐quality genome assembly. Such completeness enhances the precision of trait mapping (e.g., GWAS, QTL), enabling more accurate association between genetic loci and agronomic traits, which in turn facilitates targeted breeding strategies (Garg et al., 2024). In the “Kabusa” genome, we identified the telomeres and putative centromeric positions for all 40 chromosomes, revealing notable putative shifts in centromere positions between some homologous chromosomes. These shifts can profoundly affect chromosome behavior during meiosis and mitosis (Lu & He, 2019). The intricate relationship between centromere dynamics, genomic rearrangements, and their influence on trait variation has been well‐documented in various species (Gent et al., 2015; Han et al., 2009; Taagen et al., 2020). In the “Kabusa” genome, we also detected several para‐ and pericentric SVs. Consistent with our findings, a paracentric inversion on linkage group 16 (corresponding to DalaChr16) in “Kabusa” has been previously identified (Ehounou et al., 2022). Their study further demonstrated that this SV reduces fertility and recombination rates in “Kabusa.” The prevalence and impacts of such SVs on *D. alata* breeding programs warrant further investigation. Overall, we anticipate that the high‐quality “Kabusa” genome will play a pivotal role in accelerating greater yam improvement by providing a robust foundation for functional genomics and targeted breeding strategies.

The sex chromosomes of *D. alata* are young and at the early stage of differentiation, features observed in other plant species such as willows, poplars, strawberry, and silene (*Silene otites* and *Silene pseudotites*) (Cauret et al., 2022; Hou et al., 2015; Martin et al., 2019; Pucholt et al., 2017). In addition to the pericentric inversion, the Y chromosome exhibits a slight size reduction and a shift in putative centromere position compared to the X chromosome. Cormier et al. (2019) demonstrated that *D. alata* sex chromosomes still undergo recombination, albeit at a significantly reduced rate. This reduced recombination can impact the detection of quantitative trait loci (QTL) on these chromosomes. Genes located on sex chromosomes, particularly those linked to important agronomic traits, present both challenges and opportunities for crop breeding (Charlesworth & Harkess, 2024). To date, only a single minor QTL associated with tuber oxidative ratio has been identified on DalaChr6 (Dossa et al., 2024; Ehounou et al., 2022). It is likely that the reduced recombination rate on DalaChr6 obscures the detection of additional QTL present on this chromosome.

The collinearity of sex chromosomes in related species offers valuable insights into their evolutionary dynamics (Zhou et al., 2024). Our analysis suggests that sex chromosome formation in *D. alata* occurred after its divergence from the *D. rotundata* lineage. In *D. rotundata* (ZW system), a relatively small sex‐determining region (SDR) of approximately 161 Kb containing only three genes has been identified (Tamiru et al., 2017). In contrast, the SDR in *D. alata* spans 7.6 Mb and harbors 231 genes, reflecting a markedly different genomic architecture associated with sex determination. These differences in sex determination systems, as well as in SDR size and structure, indicate a clear case of sex chromosome turnover between the two species. Notably, *D. rotundata* frequently displays sex lability and includes monoecious cultivars (Denadi, 2022; Denadi et al., 2020), whereas such reproductive flexibility has not been reported in *D. alata* to date. Given that yams display diverse sex determination systems, including XY, ZW, and XO (*D. reticulata* and *D. sinuata*) (Sugihara et al., 2024), the *Dioscorea* genus represents an excellent model for studying sex chromosome evolution and turnovers. However, high‐quality genome resources of heterogametic sex, like the “Kabusa” genome, are essential to enable accurate detection of sex chromosomes, SDR, and sex‐determining genes.

We specifically investigated the SDR of *D. alata*, which corresponds to a large pericentric inversion on the Y chromosome and is composed of multiple inversion‐derived evolutionary strata. Our results perfectly match reports in a wide range of animals and plants, where suppression of recombination between sex chromosomes is thought to occur progressively, often mediated by chromosomal inversions. This leads to the formation of evolutionary strata, marked by distinct levels of sequence divergence between the sex chromosomes (Charlesworth et al., 2005; Hoffmann & Rieseberg, 2008; Olito & Abbott, 2023; She et al., 2023; Wang et al., 2024). The *D. alata* SDR harbors sex‐limited, sex‐specific, and sex‐biased genes important for sex phenotype expression. Many of these SDR‐associated genes are predicted to have functions related to flower organ development. This aligns with previous findings that male/female sterility and sex determination are closely tied to processes such as growth and development, pollen viability and mobility, ovule and embryo sac formation, and other gametogenic processes (Renner & Müller, 2021), as well as hormone signaling pathways (Golenberg & West, 2013). The convergent model of dioecy in plants involves a cascade of regulatory pathways, with sex‐regulatory genes playing central roles in determining sex (Zhang, Pan, et al., 2022). Although our RNA‐seq analysis identified numerous sex‐biased genes associated with the SDR, flower buds were sampled at Day 5 after blooming and therefore may not capture the earliest transcriptional events underlying sex determination. Future studies combining temporal transcriptome profiling across earlier developmental stages with detailed histological characterization will be required to identify the primary regulators initiating sex differentiation. Nonetheless, the candidate sex‐determining genes identified in *D. alata* provide a valuable foundation for advancing functional genomics and offer promising targets for future efforts in sex manipulation and breeding in this species.

## METHODS

### Plant material

*Dioscorea alata* cultivars “Kabusa” and “74F” were obtained from our germplasm collection of the Centre de coopération Internationale en Recherche Agronomique pour le Développement (CIRAD), Guadeloupe. Tubers of “Kabusa” and “74F” were planted at the CIRAD research station located at Roujol (16°10′ 56′′ N, 61° 35′ 24′′ W, 10 meters above sea level), Guadeloupe in May 2022. We also planted 10 offspring (“A36,” “A94,” “A113,” “A74,” “A96,” “A117,” “A88,” “A111,” “A64,” “A89”) from the cross “Kabusa” × “74F” (Cormier et al., 2019). Genotyping data from 37 and 108 *D. alata* accessions were retrieved from the population studied by Mota et al. (Mota et al., 2024).

### Library construction and sequencing

During vegetative stage, young leaves of “Kabusa” were collected for high‐quality genomic DNA extraction using the cetyl trimethyl ammonium bromide method (Porebski & Baum, 1997). DNA quality and concentration were tested by 0.75% agarose gel electrophoresis, NanoDrop One spectrophotometer (NanoDrop Technologies, Wilmington, DE, USA) and Qubit 3.0 Fluorometer (Life Technologies, Carlsbad, CA, USA). After the DNA quality and integrity were tested, it was randomly sheared by Covaris ultrasonic disruptor. DNBSEQ sequencing pair‐end libraries were prepared using VAHTS® Universal Plus DNA Library Prep Kit for MGI V2 (Vazyme, Nanjing, China). For Oxford Nanopore sequencing, the libraries were prepared using the SQK‐LSK110 ligation kit according to the standard protocol. The purified library was loaded onto primed R9.4.1 Spot‐On Flow Cells and sequenced using a PromethION sequencer (Oxford Nanopore Technologies (ONT), Oxford, UK) with 72‐h runs. For PacBio sequencing, the libraries were prepared using the SMRTbell Prep Kit 3.0 (Pacific Biosciences, Menlo Park, CA, USA) according to the standard protocol. The purified library was loaded and sequenced using REVIO Polymerase Kit (Pacific Biosciences, Menlo Park, CA, USA) and REVIO sequencing plate. For Hi‐C sequencing, nuclear DNA collected from young leaves was first cross‐linked to capture DNA fragment interaction and then digested with a restriction enzyme, Dpn II. Next, sequencing libraries were constructed based on the biotinylated and ligated DNA fragments and subsequently sequenced on Illumina NovaSeq 6000 (San Diego, CA, USA). Mixed tissues (flower, young leaf, old leaf, vine, tuber flesh, tuber skin, root, and petiole) of “Kabusa” were collected at different developmental stages and used for ONT full‐length Iso‐seq sequencing. The preparation of long‐read cDNA libraries was initiated using the ONT PCR‐amplified cDNA kit (SQK‐PCS109). Specifically, full‐length cDNA libraries were constructed from poly(A) + mRNA using the cDNA‐PCR Sequencing kit (SQK‐PCS109). Subsequently, the cDNA was PCR‐amplified for 13–14 cycles with barcoded adapters from the Oxford Nanopore PCR Barcoding kit (SQKPBK004). Finally, the 1D sequencing adapter was ligated to the cDNA prior to loading onto an R9.4.1 FLO PRO002 flow cell for sequencing on a PromethION platform. Flower buds were chosen at Day5 because they are RNA‐rich and at a stage when a clear developmental divergence between male and female is not yet observed (Figure 4a). Samples from 10 offspring and their parents (“Kabusa” × “74F”) were collected in triplicate and used for RNA‐seq. Total RNA was isolated from young flower buds using the E.Z.N.A.® Plant RNA Kit (Omega Biotek, Norcross, GA, USA) according to the manufacturer's instructions. Quality, purity, and quantity of the isolated RNA was checked on Agarose electrophoresis, Nanodrop (NanoDrop Technologies, Wilmington, DE, USA), and Qubit 3.0 Fluorometer (Life Technologies, Carlsbad, CA, USA), respectively. The libraries were prepared using the NEBNext® UltraTM Kit of Illumina® according to the manufacturer's instructions. RNA sequencing was conducted on Illumina Novaseq 6000 (San Diego, CA, USA).

### Genome assembly

The k‐mer depth distribution was calculated with Jellyfish (v2.2.10) (Marçais & Kingsford, 2011) based on the DNBSEQ short reads, and GCE (v1.0.0) (Liu et al., 2013) was used to estimate the genome size and heterozygosity. Four preliminary genome assemblies were generated:

1. with nextDenovo (v2.5.0, parameters: read_cutoff = 1 k, blocksize = 1 g, nextgraph_options = −a 1) (Hu et al., 2024), we performed the preliminary assembly of ONT sequencing data
2. HiFi data was assembled with hifiasm, v0.16.1‐r375 (Cheng et al., 2021)
3. A mixed assembly of HiFi and ONT ultra‐long data using hifiasm, v0.18.2‐r467
4. A mixed assembly of HiFi and Hi‐C data using hifiasm, v0.19.5‐r587

The quality of the four assemblies was evaluated and the “HiFi + Hi‐C” preliminary assembly version was selected as the foundation for the subsequent near‐complete genome assembly.

Contig sequences from the assembly were clustered into 40 chromosomes by the agglomerative hierarchical clustering method and then oriented using the ALLHiC (v0.9.8) (Zhang et al., 2019). We convert the pairwise interactions between contigs into specified binary files (.hic files) using 3D‐DNA (v180419) (Dudchenko et al., 2017) and juicer (v1.6) (Durand et al., 2016). We further refined the ordered and oriented contigs (.assembly) using Juicebox (v1.11.08) (Durand et al., 2016) to manually adjust into a reviewed assembly (.review.assembly). Gap regions were represented with 100 Ns to obtain chromosome‐level genome sequences. The Hi‐C heatmaps were generated based on contig interaction strength and genomic positions using HiCExplorer (v3.6) (Wolff et al., 2020).

To proceed to the extension and repair of telomere sequences, we employed Winnowmap (v2.03, parameters: k = 15, ‐MD) (Jain et al., 2020). All ONT reads were aligned to the genome and reads aligning once within 50 bp from the chromosome ends were collected. Occurrence of telomere repeat motifs (CCCTAAA/TTTAGGG) was calculated in each read, and we defined the read with the highest occurrence as the reference and others as query. With medaka_consensus (v1.2.1, parameters: ‐m r941_min_high_g360), we reassembled the reference telomere reads and query telomere reads to obtain the consensus sequence. Thereafter, the consensus telomere sequences were aligned to each chromosome with nucmer (v3.1) (Kurtz et al., 2004), and we replaced the telomere sequence at the chromosome ends with the best alignment result.

The next step consists of gap‐filling. Winnowmap (v2.03, parameters: k = 15, ‐MD) (Jain et al., 2020) was used to align gap‐filling data (excluding Ns) to the genome gap regions (including Ns). The priority for gap‐filling data was as follows: preliminary assembly genome versions > ONT data (consensus.fa) > HiFi data (ccs.fasta). When the alignment spans exactly across both ends of the gap, the best alignment region with the longest length was chosen to replace the gap‐containing sequence on the genome using gap‐filling data. After the gap‐filling is completed, the replaced regions in the gap‐filled genome were aligned with other assembly versions/ONT reads/HiFi reads to verify the reliability of the gap‐filling results.

### Genome evaluation

Genome assembly continuity was assessed by locating the gaps on the genome. For genome assembly consistency, the clean short reads were aligned to the genome using bwa (v0.7.17‐r1188) while ONT and HiFi reads were mapped to the genome using minimap2 (v 2.17‐r941) (Li, 2016). Alignment and coverage rates were calculated. Genome completeness was assessed using BUSCO protein mode with embryophyta_odb10 dataset (v 5.3.0; parameters: ‐‐evalue 1e‐05 ‐‐augustus) (Manni et al., 2021). Likewise, genome quality and accuracy were evaluated using Meryl (v1.3) (Rhie et al., 2020). We employed MUMmer (v4.0.0) (Marçais et al., 2018) to align the “Kabusa” haplotype genome assemblies to the female genome Dala_V2 (Bredeson et al., 2022) to determine the chromosome numbering and orientation. Then, we used SyRI (v1.6) (Goel et al., 2019) to identify collinear regions and variations between genomes.

### Repeat sequence annotation

The same pipeline was applied for HaplotypeA and HaplotypeB. First, the repeat sequences were identified and annotated. Model sequences were predicted based on the genome sequence using RepeatModeler (v2.0.4, parameters: ‐database mydb ‐threads 16, https://github.com/Dfam‐consortium/RepeatModeler). Additionally, LTR sequences were predicted using LTR_FINDER (version: Official release of LTR_FINDER_parallel, parameters: ‐threads 16 ‐harvest_out ‐size 1 000 000 ‐time 300) (Xu & Wang, 2007) and LTRharvest (v1.62, parameters: ‐minlenltr 100 ‐maxlenltr 7000 ‐mintsd 4 ‐maxtsd 6 ‐motif TGCA ‐motifmis 1 ‐similar 85 ‐vic 10 ‐seed 20 ‐seqids yes) (Ellinghaus et al., 2008). We used LTR_retriever (v2.9.0, parameters: ‐threads 16 ‐noanno) (Ou & Jiang, 2018) to remove redundancy from the sequences predicted by the two methods to obtain nonredundant LTR sequences and merged these two sets of sequences to create a *de novo* repeat sequence library. TEclass (v2.1.3, default parameters) (Abrusán et al., 2009) was employed to redefine the content labeled as “Unknown.” Then, we merged the RepBase library (v20181026) with the *de novo* library. We also used the RepeatMasker subprogram RepeatProteinMask (v4.1.5, parameters: ‐noLowSimple ‐pvalue 0.0001) to predict TE proteins. Finally, all repetitive prediction results were merged, and redundancy was removed to obtain the final collection of repetitive sequences in the “Kabusa” haplotype assemblies. Tandem repeat sequences were predicted using TRF software (v4.09, parameters: 2 7 7 80 10 502 000 ‐d ‐h) (Benson, 1999) and MISA software (v2.1, default parameters) (Beier et al., 2017).

### Gene annotation

Protein‐coding gene models were predicted by integrating three strategies: *ab initio* prediction, homology‐based search, and gene expression‐based prediction.

For gene expression‐based prediction, (a) NGS RNA‐seq clean reads were aligned to the genome using hisat2 (v2.2.1, parameters: –dta ‐p 16), and the resulting BAM files were used by stringtie (v2.2.1, parameters: ‐p 16) (Kovaka et al., 2019) to reconstruct the transcripts; (b) ONT Iso‐seq data were filtered using NanoFilt (v2.8.0, parameters: ‐l 50 ‐q 7) and full‐length sequence identification performed with pychopper (v2.7.5, parameters: ‐t 16). The obtained full‐length sequences were aligned to the “Kabusa” haplotype genomes using minimap2 (v2.26‐r1175, parameters: minimap2 ‐t 16 ‐ax splice ‐uf ‐‐secondary = no) (Li, 2016). The alignment results in BAM files were used by stringtie (v2.2.1, parameters: ‐p 16 ‐R ‐L) (Kovaka et al., 2019) to reconstruct the transcripts. (c) The transcripts predicted by the two methods were merged using tama (v1.0, parameters: ‐f filelist.txt ‐p merge, https://github.com/GenomeRIK/tama). The merged transcript results were then used with TransDecoder (v5.7.0, parameters: TransDecoder.Predict –retain_long_orfs_length 150, TransDecoder.LongOrfs ‐m 50, https://github.com/TransDecoder/TransDecoder) to predict open reading frames and finally obtain predicted coding genes.

For homology‐based search, homologous protein sequences from *D. alata* female genome (Bredeson et al., 2022), *D. rotundata* (Tamiru et al., 2017), *D. dumetorum* (Siadjeu & Albach, 2018), *D. zingiberensis* (Li et al., 2022), and *T. zeylanicus* (Vadakkemukadiyil Chellappan et al., 2019) were aligned to the “Kabusa” haplotype genomes with tblastn (v2.13.0, parameters: default) (Camacho et al., 2009) to obtain candidate regions. Then, Exonerate (v2.4.0, parameters: –model protein2genome –showtargetgff 1) (Slater & Birney, 2005) was used to predict transcripts and coding regions based on the alignment results.

The *ab initio* prediction was conducted using Augustus (v3.5.0, parameters: –uniqueGeneId = true –noInFrameStop = true –gff3 = on –strand = both) (Stanke et al., 2008) and glimmerhmm (v3.0.4, parameters: ‐f ‐g) (Delcher et al., 2007).

All the predicted gene sets were integrated using MAKER2 (v3.01.03, default parameters) (Holt & Yandell, 2011). The completeness of the predicted annotation sets was assessed using BUSCO protein mode with the Embryophyta_odb10 dataset (Manni et al., 2021). Functional annotation of protein‐coding genes was performed based on existing databases such as KEGG, GO and Uniprot. Concerning non‐coding RNAs prediction, tRNAscan‐SE (v2.0.12, parameters: ‐E ‐j tRNA.gff ‐o tRNA.result ‐f tRNA.struct –thread 16) (Chan et al., 2021) was used to find tRNA sequences in the genome, RNAmmer (v1.2, parameters: ‐S euk ‐m tsu,lsu,ssu) (Lagesen et al., 2007) was used to predict rRNA, and INFERNAL (v1.1.4) (Nawrocki & Eddy, 2013) was employed to search for ncRNA in the genome based on Rfam database (v14) (Kalvari et al., 2021).

### Centromere detection analyses

We used two complementary approaches to predict centromere regions.

1. Tandem repeat density and gene coverage approach: This method leveraged tandem repeat (TR) density and low gene density, as described in previous studies (Deng et al., 2022; Song et al., 2021). Using BEDTools (v2.30.0) (Quinlan & Hall, 2010), we calculated TR and gene coverage in 50 Kb windows across the “Kabusa” genome, visualizing continuous regions of high TR and low gene density as potential centromeric regions.
2. TR cluster (TRC)‐based approach: Following the methods used in T2T kiwifruit and *Rhodomyrtus tomentosa* genomes (Han et al., 2023; Li et al., 2023), TR sequences were identified using TRF (v4.09.1, parameters: 2 7 7 80 10 50 500 ‐d ‐h) (Benson, 1999) and clustered into TRCs using cd‐hit (v4.8.1, parameters: ‐c 0.9 ‐n 5 ‐d 0 ‐g 1 ‐aS 0.9, https://github.com/weizhongli/cdhit/releases). The most abundant TRCs were plotted to aid centromere identification.
3. Final prediction and visualization: We used the longest TRC as the representative sequence and minimized overlaps using RepeatMasker. BEDTools (v2.30.0) (Quinlan & Hall, 2010) was then used to merge and define centromere regions, which were visualized with RIdeogram (Hao et al., 2020) and LINKVIEW2 (v1.0.5, https://github.com/YangJianshun/LINKVIEW2), alongside features like rDNA and telomeres.

### Sequence variation between haplotype genomes of “Kabusa” and evolutionary analysis with other *Dioscorea* species

Haplotype genomes of “Kabusa” were aligned using MUMmer (v4.0.0rc1; default parameters) (Marçais et al., 2018), with HaplotypeA as the reference. Then, variants were detected using SyRI (v1.5; default parameters) (Goel et al., 2019). For synteny analysis between haplotype genomes of “Kabusa” and the female *D. alata* genome (Bredeson et al., 2022; Zhang et al., 2025), gene sequences were aligned using LAST (v1170) (Frith et al., 2010) to identify similar gene pairs. These gene pairs were then analyzed using JCVI (v0.9.13) (Tang et al., 2024) to determine if they are adjacent on the chromosomes based on annotation files (gff3), thereby identifying all genes in syntenic blocks. The synteny plots were generated using SyRI (v1.5; default parameters) (Goel et al., 2019).

To identify shared and unique genes between the two haplotype genomes (HaplotypeA and HaplotypeB), the genomes were clustered into gene families using Orthofinder (v2.3.12) (Emms & Kelly, 2019). Protein sequences from HaplotypeA were extracted and compared to the HaplotypeB genome using tblastn (v2.13.0) (Camacho et al., 2009). Structural integrity of matching genes was verified using gffread (v0.12.7). Protein sequences of structurally complete genes were further compared to HaplotypeA's proteins using blastp (v2.6.0) (Camacho et al., 2009). Genes with alignment scores >150 were deemed homologous and removed from HaplotypeA's unique gene list. The remaining genes were considered unique to HaplotypeA. From the unique gene list of HaplotypeA, genes located on Chr6 were identified as DalaChr6A‐specific unique genes. The same process was applied to identify unique genes in HaplotypeB and DalaChr6B‐specific unique genes.

For phylogenetic tree construction between *Dioscorea* species, a multiple sequence alignment of protein sequences for each single‐copy gene family was performed using MUSCLE (v3.8.31) (Edgar, 2004). The alignment results were then filtered using trimAl (v1.2rev59; parameters: ‐gt 0.2) (Capella‐Gutiérrez et al., 2009). The filtered alignments were concatenated into the supergene. A maximum likelihood phylogenetic tree was constructed based on the supergene using RAxML (v8.2.10) (Stamatakis, 2014) with the PROTGAMMAWAG model. Divergence times for the selected species (*D. alata*, *D. rotundata*, *D. tokoro*, and *D*. *zingiberensis*) were estimated using the mcmctree program from the PAML package (v4.9; parameters: nsample = 3 000 000; burnin = 8 000 000; seqtype = 0; model = 4) (Yang, 2007). A synonymous substitution rate of 4 × 10^−9^ substitutions per site per year, previously estimated for *Dioscorea*, was used for molecular dating (Hu et al., 2025).

### Transcriptome analysis

Newly generated raw reads from flower buds, along with short reads from mature tubers of three female *D. alata* accessions (“Dou,” “Kinabayo,” and “74F”) and three male accessions (“Divin,” “Plimbite,” and “Toufi‐Tetea”), previously generated by Mota et al. (Mota et al., 2024) (PRJNA918625), were used to perform a comparative gene expression analysis between male and female groups. The package fastp (v0.21.0) (Chen, 2023) was used to filter raw reads (150 bp paired‐end) and discard low‐quality reads. Several libraries were found to be of poor quality or contaminated after sequencing; therefore, we excluded them from further analyses. The cleaned reads from each sample were mapped to the haploid consensus genome of the “Kabusa” genome (specifically the HapA assembly, which includes the Y chromosome + the HXR) using STAR (v2.7.9, parameters:default) (Dobin et al., 2013). This choice was made to enable a focused analysis of male‐specific and shared gene expression. The RSEM package (Li & Dewey, 2011) was used to calculate gene expression level for each sample expressed as fragments per kilobase of transcript per million fragments mapped. The edgeR (v3.32.1) software (Robinson et al., 2010) was used for differential expression analysis. Selection thresholds of FDR <0.05 and |log_2_ FC| > 1. Functional enrichment analysis was performed using the R package clusterProfiler v4.0 (Wu et al., 2021) based on the functional annotations of all genes in this species, including Gene Ontology (Ashburner et al., 2000) and Kyoto Encyclopedia of Genes and Genomes (Kanehisa & Goto, 2000).

### Refining sex‐determination region

Whole genome resequencing raw data of 20 male and 17 female accessions were downloaded from NCBI (PRJNA918625). We constructed a male‐pool by merging male data and a female‐pool by merging female data. The two pools were mapped to the HaplotypeA of the “Kabusa” genome. GATK (v4.6.1.0) (McKenna et al., 2010) was used for SNP calling and *Fst* was estimated between the two pools with VCFtools (0.1.17) (Danecek et al., 2011). Next, SNPs that are homozygous in the female pool but heterozygous in the two pools were filtered. The variant density in a 100 Kb window was calculated and plotted with the circlize package (Gu et al., 2014) in R (v4.4.2).

### Genome wide association studies

A total of 200 *D. alata* accessions were planted at Roujol and Godet research stations in Guadeloupe during seasons 2023 and 2024. Sex phenotypes were recorded during flowering period (September to December) each year. Flowering was recorded in 108 accessions including 68 males and 40 females. Genotyping data from the 108 *D. alata* accessions were retrieved from the population studied by Mota et al. (Mota et al., 2024) (PRJNA918625). SNPs and InDels calls were performed as described above using GATK (v4.6.1.0) (McKenna et al., 2010). GWAS was conducted independently for SNPs and InDels genotype matrices using PLINK v1.90b6.16 (Purcell et al., 2007) with the logistic regression model that included the top three principal components from SNPs and InDels genotype matrices. The Manhattan plots were generated in R (v4.4.2). Markers having a significant association with traits were determined by the adjusted *P* value.

### K‐mer analysis

To identify sex‐specific sequences based on k‐mer composition, we performed a k‐mer counting analysis using KMC3 (v3.2.1) (Kokot et al., 2017). Cleaned Illumina paired‐end reads from 20 male and 20 female individuals were used for this analysis. For each sample, canonical 27‐mers (k = 27) were counted independently using the following parameters: ‐k27 ‐ci5 ‐fq ‐m64 ‐t4. The minimum count threshold (−ci5) was applied to filter out low‐frequency k‐mers likely resulting from sequencing errors. Reads were processed in paired‐end mode (−fq) using compressed FASTQ files (*_clean_R1.fq.gz and *_clean_R2.fq.gz). The k‐mer databases generated for each individual were stored separately to allow downstream comparison between male and female groups. (1) Canonical k‐mer databases previously generated using KMC3 were compared using kmc_tools. Specifically, k‐mers unique to males were identified by subtracting male using kmers_subtract producing male‐specific k‐mers. (2) The unique k‐mers were dumped into text files and filtered to exclude sequences containing ambiguous nucleotides (‘N'). Cleaned lists were then converted into FASTA format using awk, with each k‐mer assigned a unique identifier. (3) Male‐specific k‐mers were mapped to the *D. alata* HaplotypeA using bwa mem, with relaxed parameters (−k 21 ‐T 21 ‐a) to accommodate short reads and allow multi‐mapping. The resulting SAM file was converted to sorted BAM format using SAMtools, (v1.18) (Danecek et al., 2021) followed by indexing for downstream visualization and coverage analysis.

### Read coverage analysis

To quantify sequencing read coverage, we used Mosdepth (v0.3.3) (Pedersen & Quinlan, 2018). A 10 Kb sliding window BED file was generated to cover DalaChr6A. BAM files were grouped by sex based on metadata and processed independently for the 68 male and 40 female samples. Coverage depth was calculated across each window using Mosdepth with the options ‐n ‐x to exclude duplicate and secondary alignments. For each window, the mean depth was calculated across all individuals of each sex using custom AWK scripts. The final dataset consisted of a merged table reporting average male and female coverage per window across the DalaChr6A.

### Sex chromosome divergence analysis

We estimated nonsynonymous (*Ka*) and synonymous (*Ks*) substitution rates for gene pairs located on the X and Y chromosomes. First, we extracted gene coordinates from specific regions of interest SDR, INV1–4, and XSR, PAR, MSY—using bedtools intersect and GFF annotations from the “Kabusa” haplotype‐resolved genome assembly. The total number of genes in each region was quantified based on gene annotations from chromosomes DalaChr6A (Y) and DalaChr6B (X). Next, their corresponding coding DNA sequences and protein sequences were retrieved using seqtk‐1.5 (r133; https://github.com/lh3/seqtk). Orthologous gene pairs between Haplotypes A and B were determined using an anchor file from whole‐genome synteny analysis. For each orthologous gene pair, protein sequences were aligned using MAFFT (v7.453) (Rozewicki et al., 2019). Codon alignments were then generated using PAL2NAL (v14) (Suyama et al., 2006). The resulting codon alignments were cleaned, concatenated, and used as input to KaKs_Calculator (v2.0) (Wang et al., 2010) with the Yang‐Nielsen model to estimate pairwise Ka, Ks, and Ka/Ks ratios.

### PACE marker development and experimental procedure

SNP genotyping was performed as described by von Maydell (von Maydell, 2023). DNA was extracted from leaf tissue of 146 *D. alata* accessions using mixed alkyl trimethylammonium bromide buffer and NucleoMag Plant Kit (Macherey‐Nagel, Düren, Germany). We used the PACE™ master mix (3CR Bioscience, United Kingdom) (standard protocol), and runs were performed on LightCycler480 (Roche, Basel, Switzerland). Five negative controls (water) were included in the experiment. From the raw fluorescence data, allele calling was performed by defining fluorescence clusters. Plotting was conducted with the R package snpclust (v1.1; https://github.com/jframi/snpclust).

### Methylation analysis

HiFi CCS reads were used for estimating the methylation frequencies of the CpGs according to the ccsmethphase pipeline developed by Ni et al. (2023). To phase the reads, we used Meryl (v1.4.1) (Rhie et al., 2020) to find unique k‐mers in the reference genome of each haplotype as described by Nie et al. (2024) and classify the reads using the unique k‐mers. The methylation frequencies were estimated for binned regions (100 Kb) in each haplotype assembly.

### Statistical analysis

All statistical analyses were performed in R (v4.4.2). Depending on the data distribution and experimental design, both parametric tests (*t*‐tests and one‐way ANOVA) and non‐parametric tests (Wilcoxon rank‐sum test and Kruskal–Wallis test) were applied. A significance threshold of *P* < 0.05 was used throughout.

## Author Contributions

Conceptualization: Komivi Dossa and Hanâ Chaïr; Methodology: Komivi Dossa, Ting Qiu, Yao Chen, Peng Ni, Xiaojun Su, Gang Chen, Petre I. Dobrev, Bertrand Fouks, Gemma Arnau, Pierre Mournet, Jianxin Wang, and Hanâ Chaïr; Software: Komivi Dossa, Ting Qiu, Yao Chen, Peng Ni, Xiaojun Su, Gang Chen, and Jianxin Wang; Formal analysis: Yedomon Ange Bovys Zoclanclounon, Muhammad Amjad Nawaz, Erick Malédon, Moussa Diouf, Petre I. Dobrev, Benjamin Karikari, Bertrand Fouks, and Pierre Mournet; Investigation: Erick Malédon, Marie‐Claire Gravillon, Christophe Perrot, Elie Nudol, Lévy Laurent, and Pierre Mournet; Resources: Komivi Dossa and Hanâ Chaïr; Data curation: Komivi Dossa, Ting Qiu, Yedomon Ange Bovys Zoclanclounon, Pierre Mournet, Yao Chen, Moussa Diouf, Peng Ni, Xiaojun Su, Gang Chen, Petre I. Dobrev, Bertrand Fouks, and Jianxin Wang; Writing—original draft preparation: Komivi Dossa, Ting Qiu, Yedomon Ange Bovys Zoclanclounon, and Muhammad Amjad Nawaz; Writing—Review and Editing: Hanâ Chaïr and Gemma Arnau; Visualization: Komivi Dossa, Ting Qiu, Gang Chen, and Muhammad Amjad Nawaz; Supervision: Hanâ Chaïr and Benjamin Karikari; Project administration: Komivi Dossa and Hanâ Chaïr; Funding Acquisition: Komivi Dossa and Hanâ Chaïr. All authors have read and agreed to the published version of the manuscript.

## Conflict of Interests

The authors declare that they have no competing interests.

## Supporting information


**Figure S1.** Flow cytometry‐based determination of the ploidy level in “Kabusa.” The ploidy level was assessed by comparing the position of the main peak with the references indicated by the arrows. According to the results, “Kabusa” is identified as a diploid cultivar (2×).

**Figure S2.** k‐mer analysis for estimation of the “Kabusa” genome size. The genome size was estimated to approximately 494.13 Mb.

**Figure S3.** Distribution of the length of gene and gene components in the “Kabusa” genome assembly compared to related species. (A) Gene length distribution. (B) Coding sequence (CDS) length distribution. (C) Exon length distribution. (D) Intron length distribution.

**Figure S4.** Centromere and telomere detection map in the two haplotype genome assemblies of “Kabusa.”

**Figure S5.** Illustrations of alignments of preliminary assemblies (A, B) or ONT reads (C) that clearly spanned gap regions providing strong evidence of the accuracy of the gap filling stage.

**Figure S6.** Centromere detection in the two haplotype genome assemblies of “Kabusa.” The first layer representing LTR/Copia density (blue color), the second layer representing LTR/Gypsy density (orange color), the third layer representing tandem repeat density (ruby red color), and the fourth layer representing the gene density (turquoise color) along the chromosomes.

**Figure S7.** Structural variation (SV) between the two haplotype assemblies of the “Kabusa” genome.

**Figure S8.** Collinearity analysis between the HaplotypeA, HaplotypeB of the “Kabusa” genome, and the female genome assembly from Bredeson et al. (Bredeson et al., 2022).

**Figure S9.** Intergenomic comparison of the HaplotypeA, HaplotypeB of the “Kabusa” genome and the female genome assembly from Bredeson et al. (Bredeson et al., 2022) Red circle shows the location of the pericentric inversion in DalaChr6A versus DalaChr6F but absent in DalaChr6B versus DalaChr6F.

**Figure S10.** IGV snapshots showing the alignment of HiFi reads spanning the breakpoints of the inverted region between the Y chromosome (DalaChr6A) and X chromosome (DalaChr6B) of the “Kabusa” genome.

**Figure S11.** Sex chromosome evolution in yams. (A) Synteny analysis between genomes of four yam species with emphasis on sex chromosomes reported to date. Dala_V2 = female genome of *Dioscorea alata*, DalA and DalB = Two haplotype assemblies of the “Kabusa” genome (*D. alata*), Drot = *D. rotundata*, Dtok = *D. tokoro* and Dzin = *D. zingiberensis*. *Dioscorea alata* and *D. rotundata* belong to the section Enantiophyllum while *D. tokoro* and *D. zingiberensis* belong to the section Stenophora. XY and ZW denote the sex determination systems reported in the yam species. Numbers before and within brackets represent divergence time million years ago.

**Figure S12.** Distribution and genome‐wide comparison of transposable elements in the two haplotype assemblies of the “Kabusa” genome. (A, B) Genome‐wide distribution of transposable element classes across the two haplotype assemblies. (C) Comparison of LTR/Gypsy and LTR/Copia coverage between the sex‐linked regions (SDR in haplotype A and HXR in haplotype B) and their respective autosomal genomic backgrounds. TE coverage was calculated in nonoverlapping 100‐kb windows.

**Figure S13.** Gene expression profiling and heatmap clustering of male and female groups.

**Figure S14.** Gene ontology enrichment analysis of the 6917 sex‐biased expressed genes detected between male and female groups in *Dioscorea alata*.

**Figure S15.** Patterns of methylation level (DNA methylation 5mC at CpG sites) along homologous chromosomes in the two haplotype assemblies of the “Kabusa” genome. Plot was generated using a window of 100 Kb.

**Figure S16.** Genotyping of *D. alata* diversity panel (146 accessions from different countries) with the *sda6* PACE marker. Fluorescence signals are plotted by accession and observed sex. In *x*‐axis, the “T” fluorescence allele (X), and in *y*‐axis, the “G” fluorescence allele (Y).

**Table S1.** Statistics of the Illumina short reads data.
**Table S2.** Statistics of k‐mer analysis based on Illumina short reads data.
**Table S3.** Oxford nanopore technology ultra‐long data statistics.
**Table S4.** Pacbio HiFi ultra‐long data statistics.
**Table S5.** Statistics of the Hi‐C data.
**Table S6.** Four assembly strategies evaluated on the quality of the contig‐level genomes.
**Table S7.** Detailed statistical results of each chromosome and its length in the hybrid assembly version (HiFi + Hi‐C).
**Table S8.** Position of telomeric repeat sequences along the chromosomes in the “Kabusa” genome.
**Table S9.** Gap filling in the genome assembly.
**Table S10.** Detailed statistical results of each chromosome and its length in the final near‐complete “Kabusa” genome assembly.
**Table S11.** Statistics of repetitive element annotation in the genome of “Kabusa.”
**Table S12.** Statistics of ONT based full‐length transcriptome data for genome annotation.
**Table S13.** Statistics of prediction, functional annotation of coding genes and noncoding RNA annotation in the HaplotypeA and HaplotypeB genome assemblies of “Kabusa.”
**Table S14.** Statistics of gene set BUSCO evaluation (busco_lineage:embryophyta_odb10) in the HaplotypeA and HaplotypeB genome assemblies of “Kabusa.”
**Table S15.** Position of centromeric repeat sequences along the chromosomes in the “Kabusa” genome.
**Table S16.** Report of the Liftoff annotation of the female genome *D. alata*_v2 based on HaplotypeA of the “Kabusa” genome.
**Table S17.** Statistics of variants detected between the two haplotype genome assemblies of “Kabusa” and their impact on gene function.
**Table S18.** PLINK logistic genome‐wide association study (GWAS) results based on SNPs and Indels markers. One hundred and two *D. alata* accessions were evaluated for sex phenotype in the field over 2 years at two sites in Guadeloupe.
**Table S19.** Genes located within the SDR and HXR and their functional annotation. INV1‐INV4 = Inversion 1‐Inversion 4. XSR = X‐specific region; MSY = Male‐Specific region of the Y chromosome.
**Table S20.** Sex‐linked genes that have functional homologs on autosomal chromosomes in the *D. alata* “Kabusa” genome.
**Table S21:** Samples used for RNA‐seq transcriptome.
**Table S22.** Statistics of RNA‐seq transcriptome data from male and female flower buds. ‐1, −2, −3 represent the three biological replicates. If a genotype does not have three replicates, it means a replicate was contaminated and was excluded.
**Table S23.** Sex‐biased genes located within the SDR and HXR in *D. alata*. Up/down expression in male compared to female flower buds.
**Table S24.** Development of a PACE sda6 marker linked to sex determination in *Dioscorea alata*. A sex‐linked locus was detected via GWAS in the gene DalaChr6AG083080 at the position 91 bp. All female offspring are homozygous TT (XX) while male are heterozygous GT (XY).
**Table S25.** Details on the 146 *D. alata* germplasm genotyped with the *sda6* PACE marker.
**Table S26.** Details on the *D. alata* germplasm used for genetic analyses.

## Data Availability

All raw high‐throughput sequencing data comprising Illumina, Oxford Nanopore, PacBio, and Hi‐C reads are deposited in NCBI under project numbers PRJNA1175864 and PRJNA880983. Whole genome assemblies are available under the Umbrella BioProject number PRJNA1182344 with accession numbers PRJNA1181019 (HaplotypeA) and PRJNA1181020 (HaplotypeB). The genome assemblies and annotation files are also available at https://yam‐genome‐hub.southgreen.fr.
