# Supplementary material for: Haplotype‐resolved genome assembly of a male Dioscorea alata cultivar reveals the structure and evolution of young sex chromosomes
Source: Plant J. 2026 Aug 17;127(4):e71091. doi: 10.1111/tpj.71091 (PMC13479938; doi:10.1111/tpj.71091)

Supplementary Figure 13: Gene expression profiling and heatmap clustering of male and female groups.

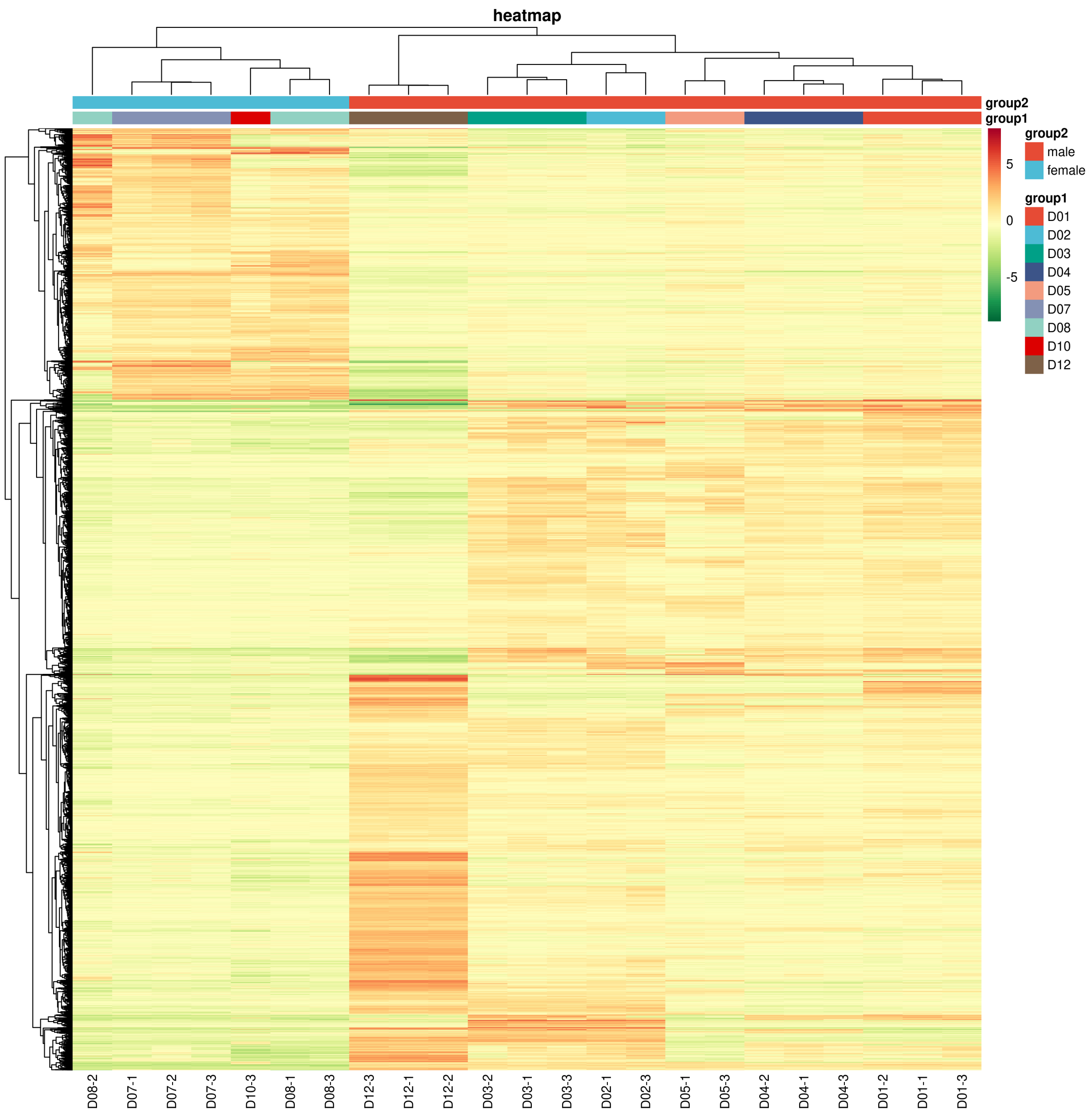

Supplement: Supplementary file 1 [file TPJ-127-0-s001.pdf]
