# Supplementary material for: Haplotype‐resolved genome assembly of a male Dioscorea alata cultivar reveals the structure and evolution of young sex chromosomes
Source: Plant J. 2026 Aug 17;127(4):e71091. doi: 10.1111/tpj.71091 (PMC13479938; doi:10.1111/tpj.71091)

Supplementary Figure 3: Distribution of the length of gene and gene components in the 'Kabusa' genome assembly compared to related species. **A.** Gene length distribution. **B.** Coding sequence (CDS) length distribution. **C.** Exon length distribution. **D.** Intron length distribution.

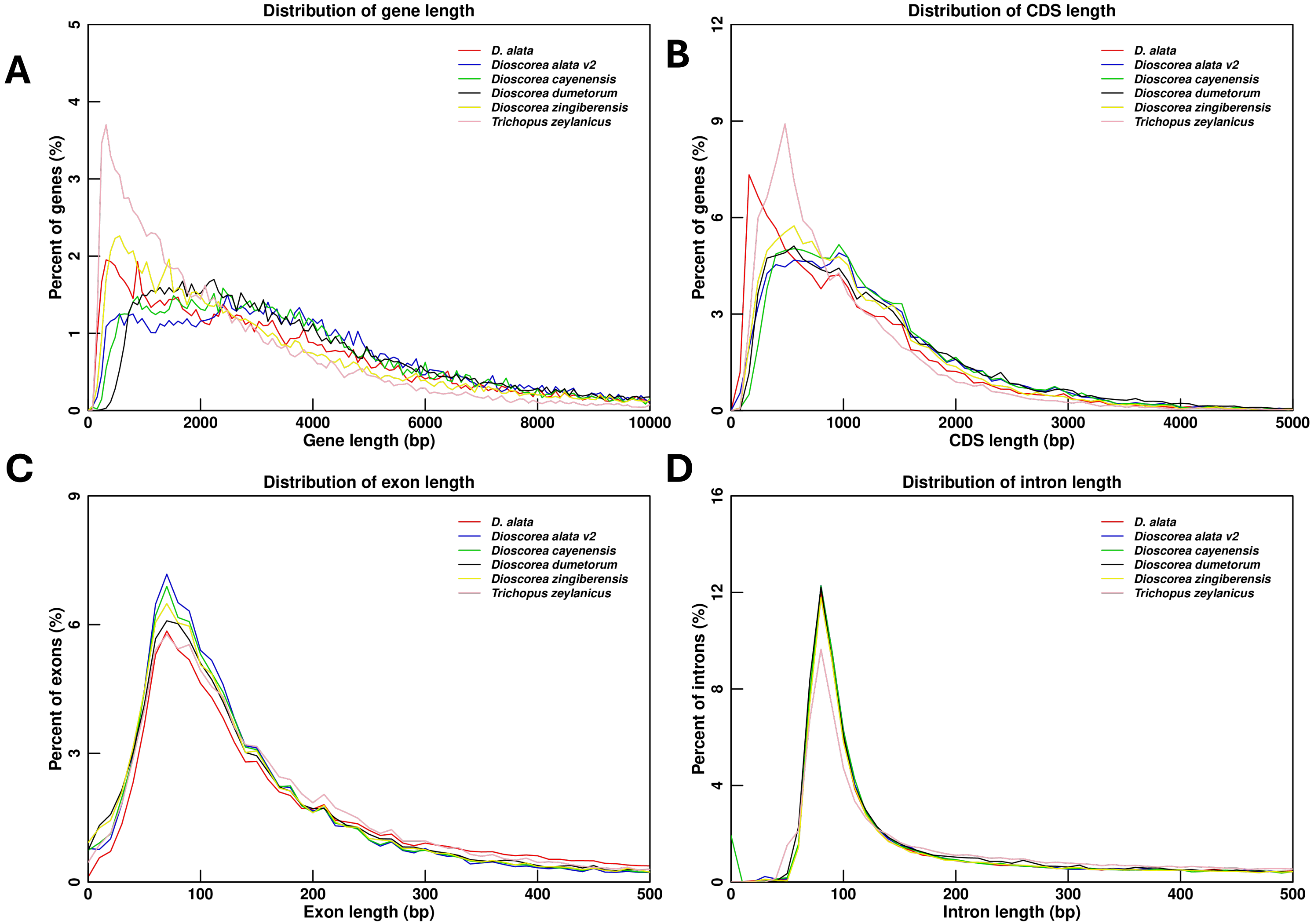

Supplement: Supplementary file 2 [file TPJ-127-0-s002.pdf]
