# Supplementary material for: Haplotype‐resolved genome assembly of a male Dioscorea alata cultivar reveals the structure and evolution of young sex chromosomes
Source: Plant J. 2026 Aug 17;127(4):e71091. doi: 10.1111/tpj.71091 (PMC13479938; doi:10.1111/tpj.71091)

Supplementary Figure 14: Gene ontology enrichment analysis of the 6917 sex-biased expressed genes detected between male and female groups in *Dioscorea alata*

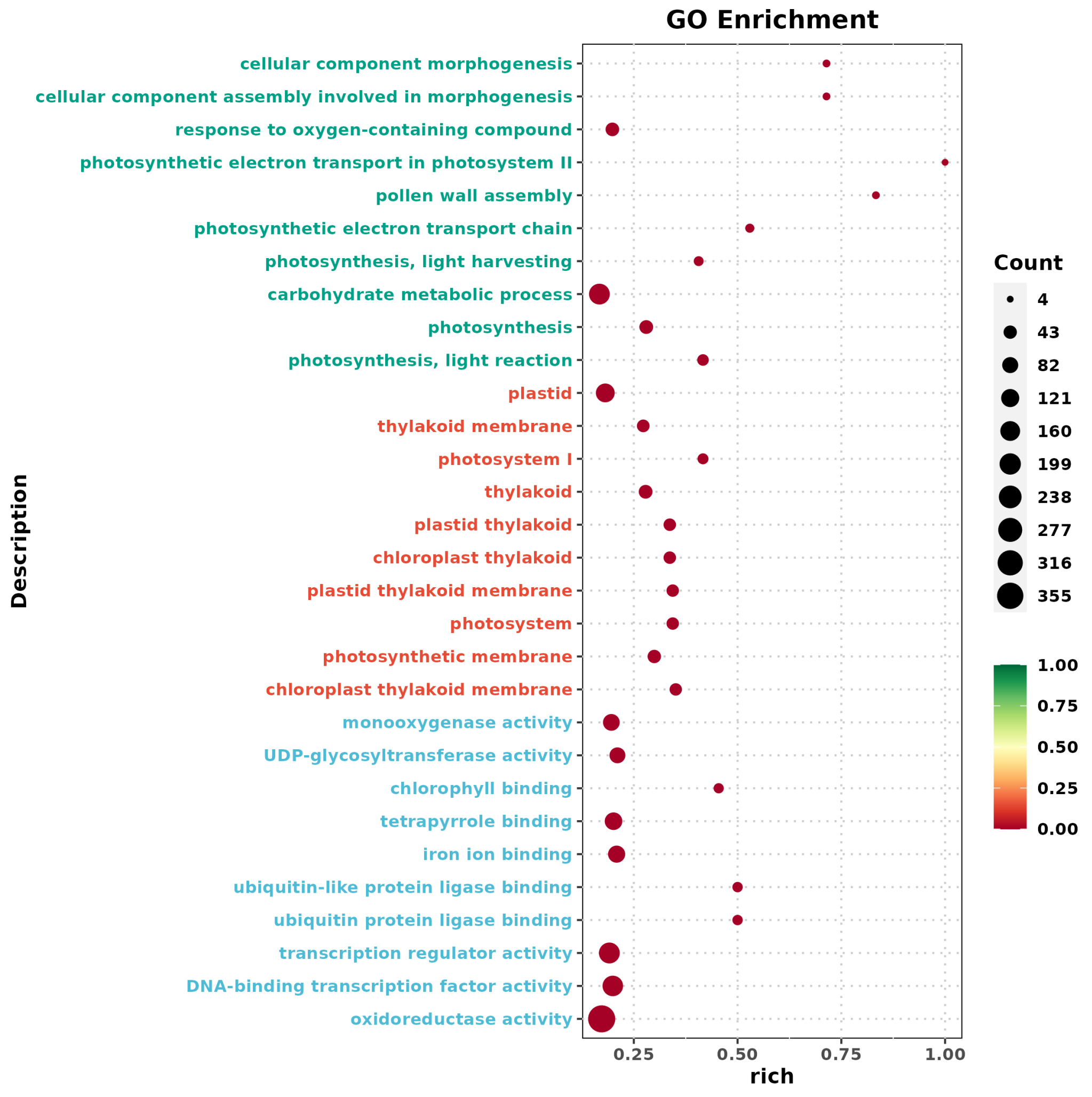

Supplement: Supplementary file 3 [file TPJ-127-0-s003.pdf]
