# Supplementary material for: Haplotype‐resolved genome assembly of a male Dioscorea alata cultivar reveals the structure and evolution of young sex chromosomes
Source: Plant J. 2026 Aug 17;127(4):e71091. doi: 10.1111/tpj.71091 (PMC13479938; doi:10.1111/tpj.71091)

Supplementary Figure 7: Structural variation (SV) between the two haplotype assemblies of the 'Kabusa' genome. **A**. Quantity of SVs mapped to different genomic regions. **B**. Gene ontology enrichment analysis of SV-influenced genes.

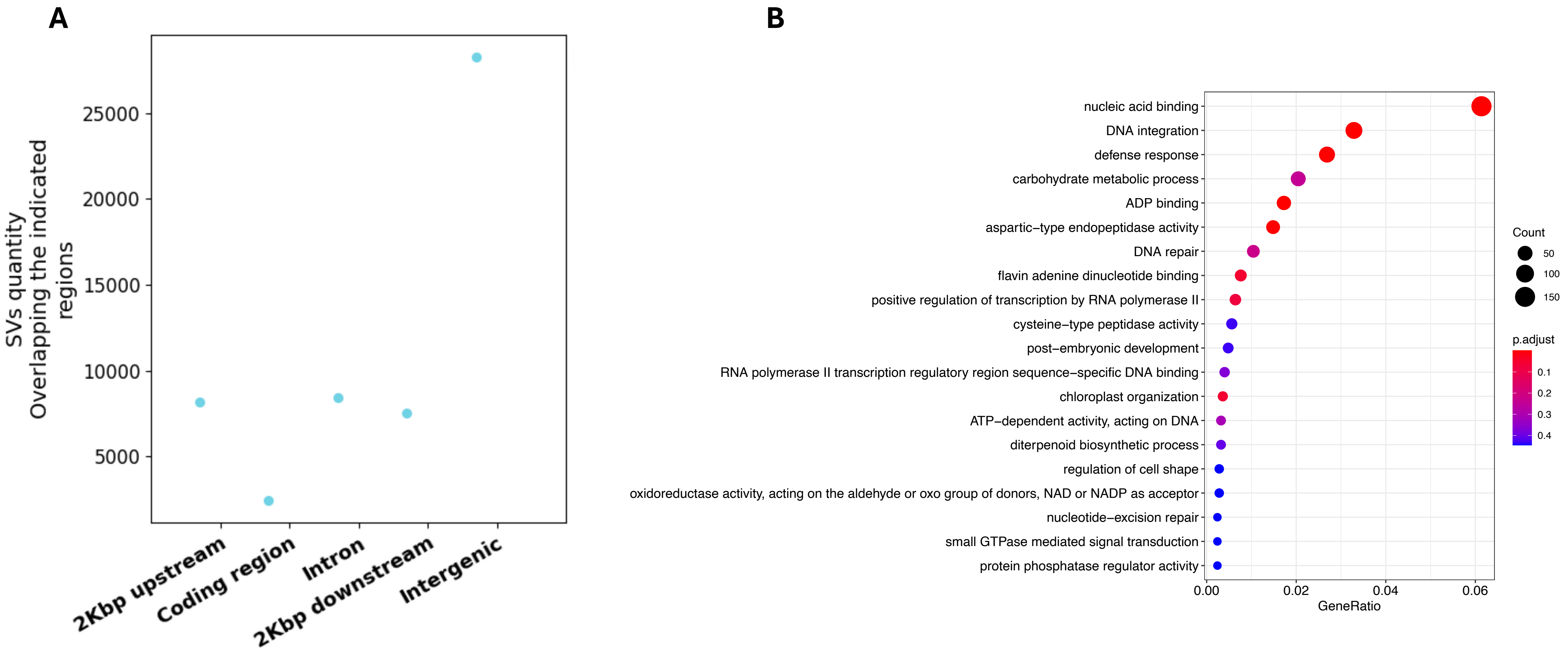

Supplement: Supplementary file 4 [file TPJ-127-0-s004.pdf]
