# Supplementary material for: Haplotype‐resolved genome assembly of a male Dioscorea alata cultivar reveals the structure and evolution of young sex chromosomes
Source: Plant J. 2026 Aug 17;127(4):e71091. doi: 10.1111/tpj.71091 (PMC13479938; doi:10.1111/tpj.71091)

Supplementary Figure 10 : IGV snapshots showing the alignment of HiFi reads spanning the breakpoints of the inverted region bewteen the Y chromosome (DalaChr6A) and X chromosome (DalaChr6B) of the 'Kabusa' genome

chr6A_1624597-1625979

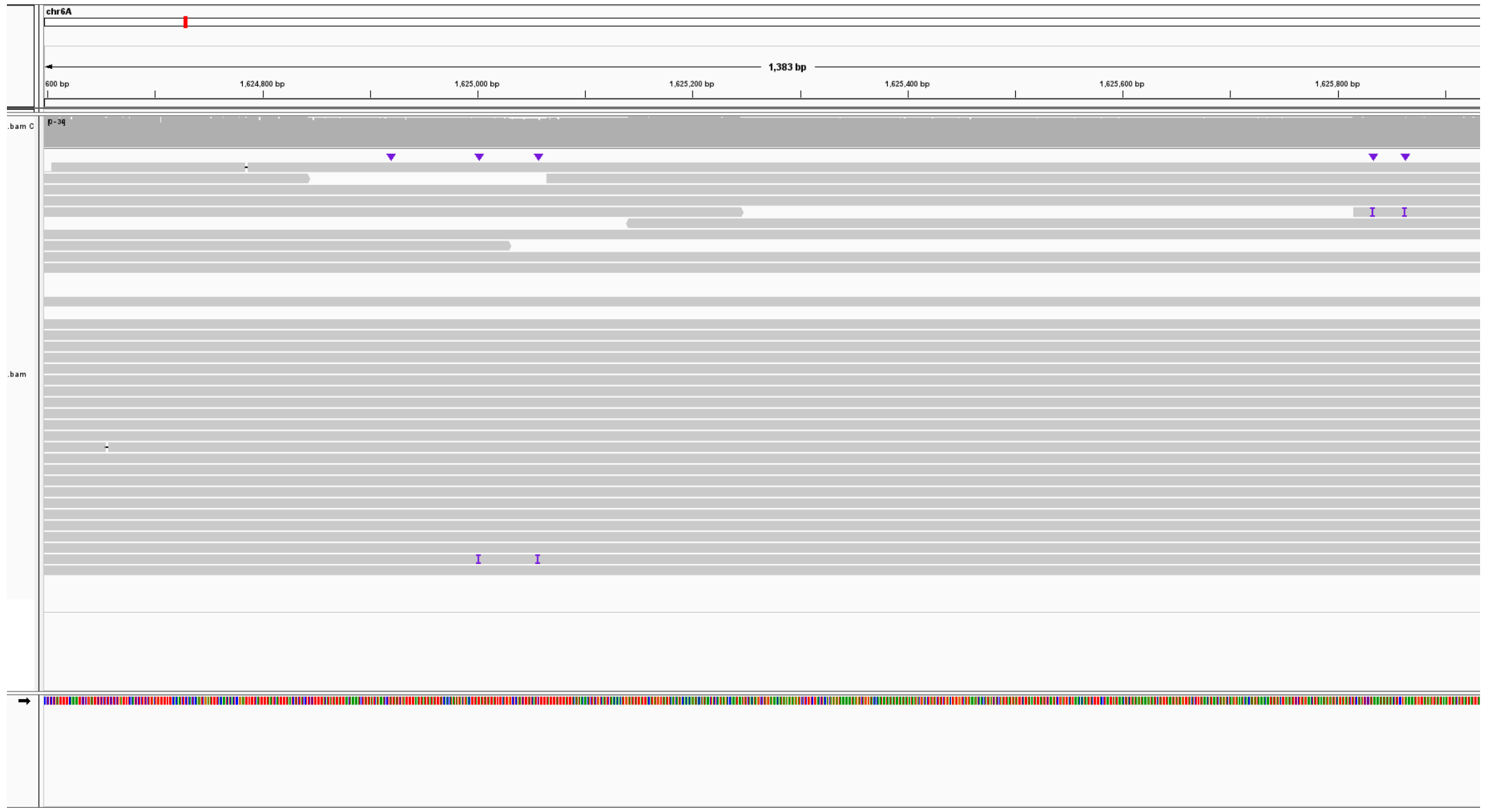

chr6A_9195788-9197170

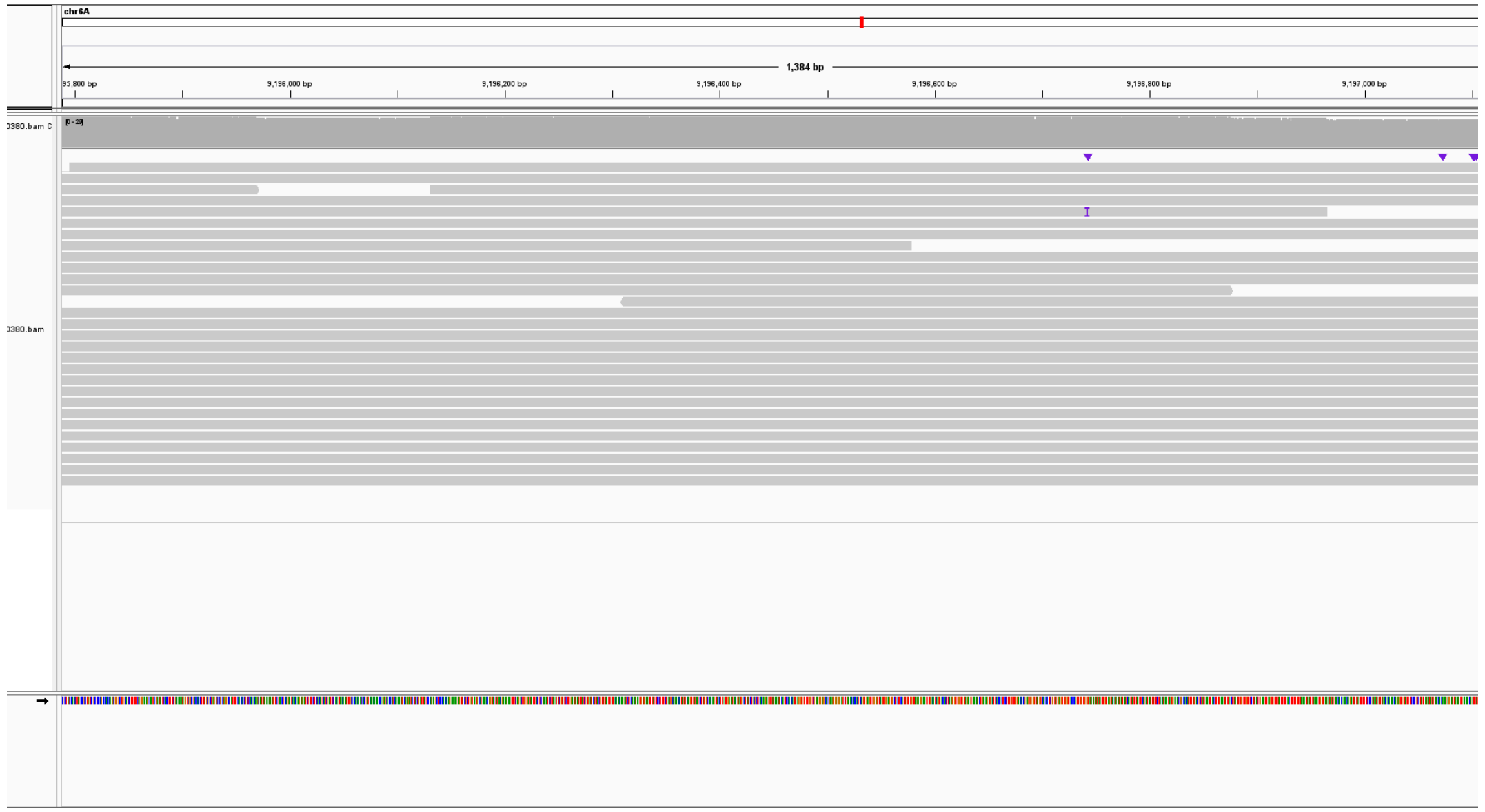

chr6B_1573680-1575092

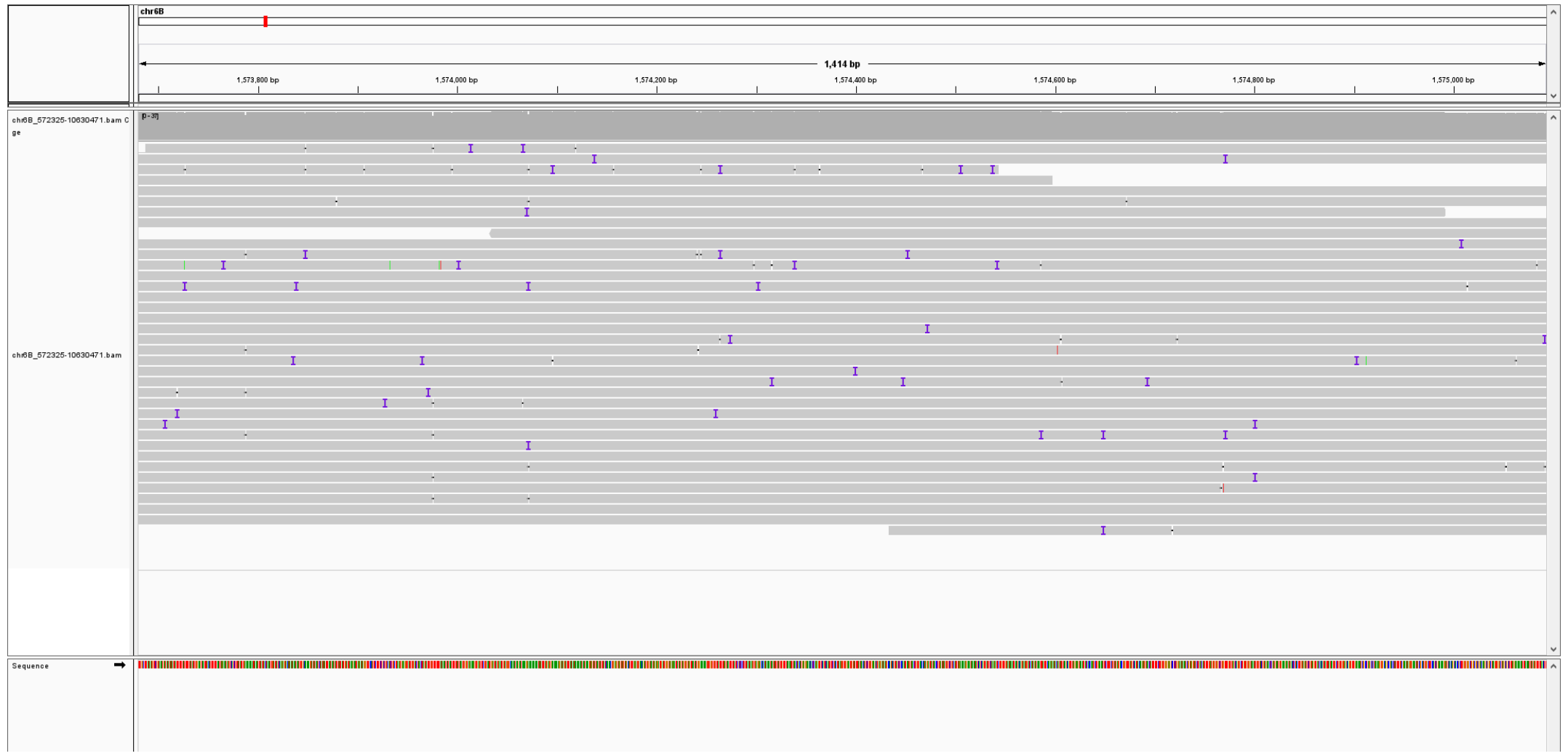

chr6B_9629770-9631182

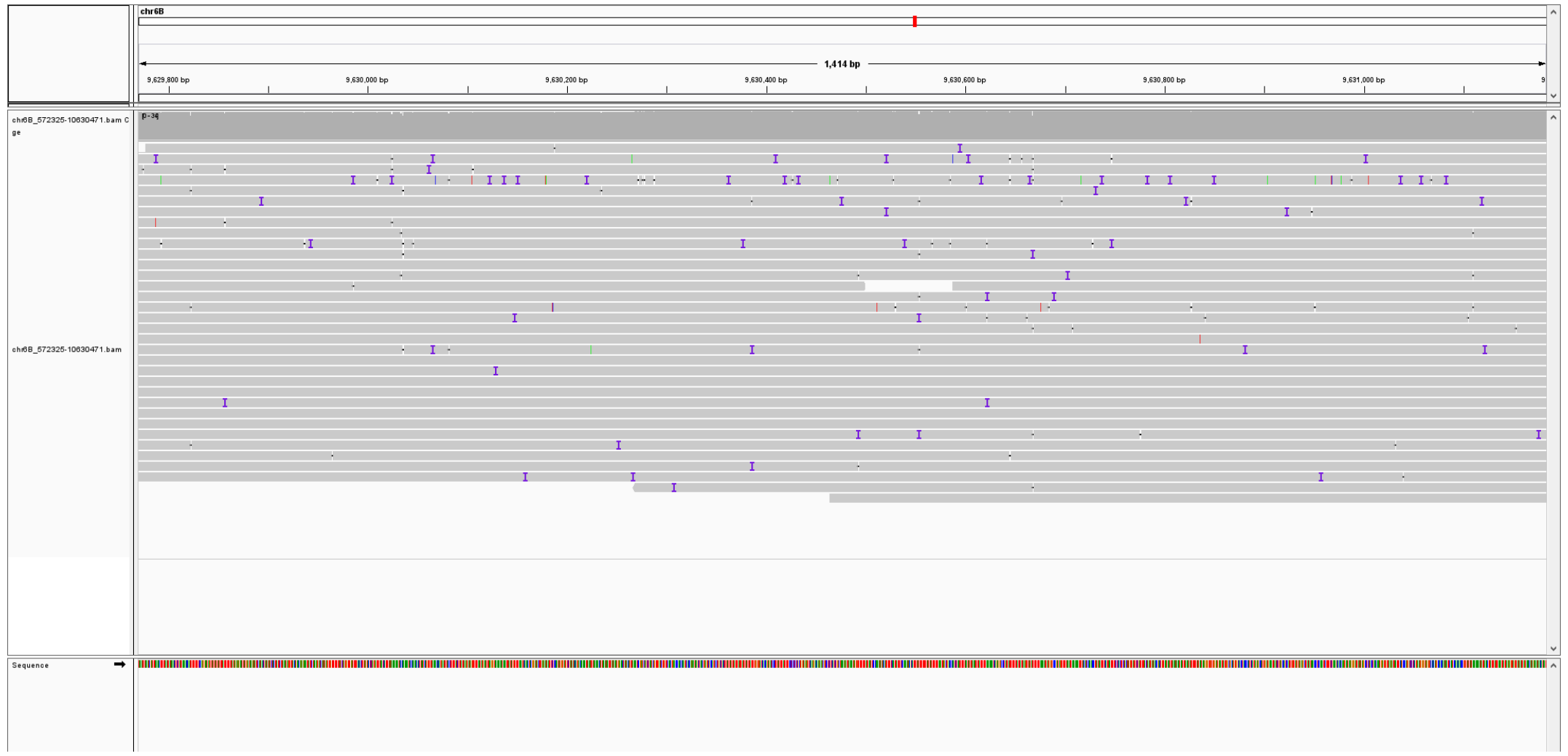

Supplement: Supplementary file 5 [file TPJ-127-0-s005.pdf]
