# Supplementary material for: Haplotype‐resolved genome assembly of a male Dioscorea alata cultivar reveals the structure and evolution of young sex chromosomes
Source: Plant J. 2026 Aug 17;127(4):e71091. doi: 10.1111/tpj.71091 (PMC13479938; doi:10.1111/tpj.71091)
Supplement: Supplementary file 6 [file TPJ-127-0-s007.pdf]

Supplementary Figure 12. Distribution and genome-wide comparison of transposable elements in the two haplotype assemblies of the 'Kabusa' genome. (A-B) Genome-wide distribution of transposable element classes across the two haplotype assemblies. (C) Comparison of LTR/Gypsy and LTR/Copia coverage between the sex-linked regions (SDR in haplotype A and HXR in haplotype B) and their respective autosomal genomic backgrounds. TE coverage was calculated in non-overlapping 100-kb windows.

(**A**)

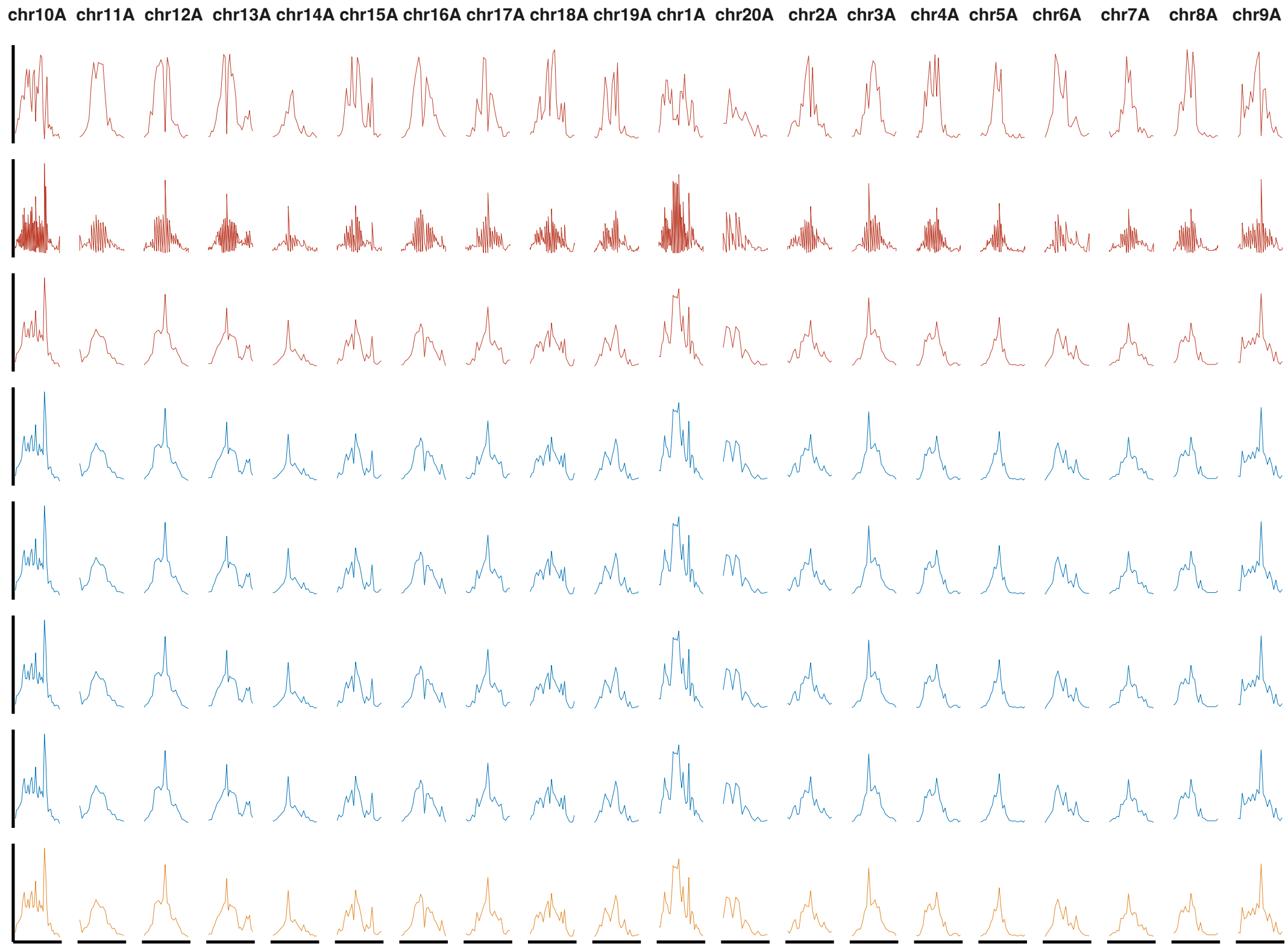

(**B**)

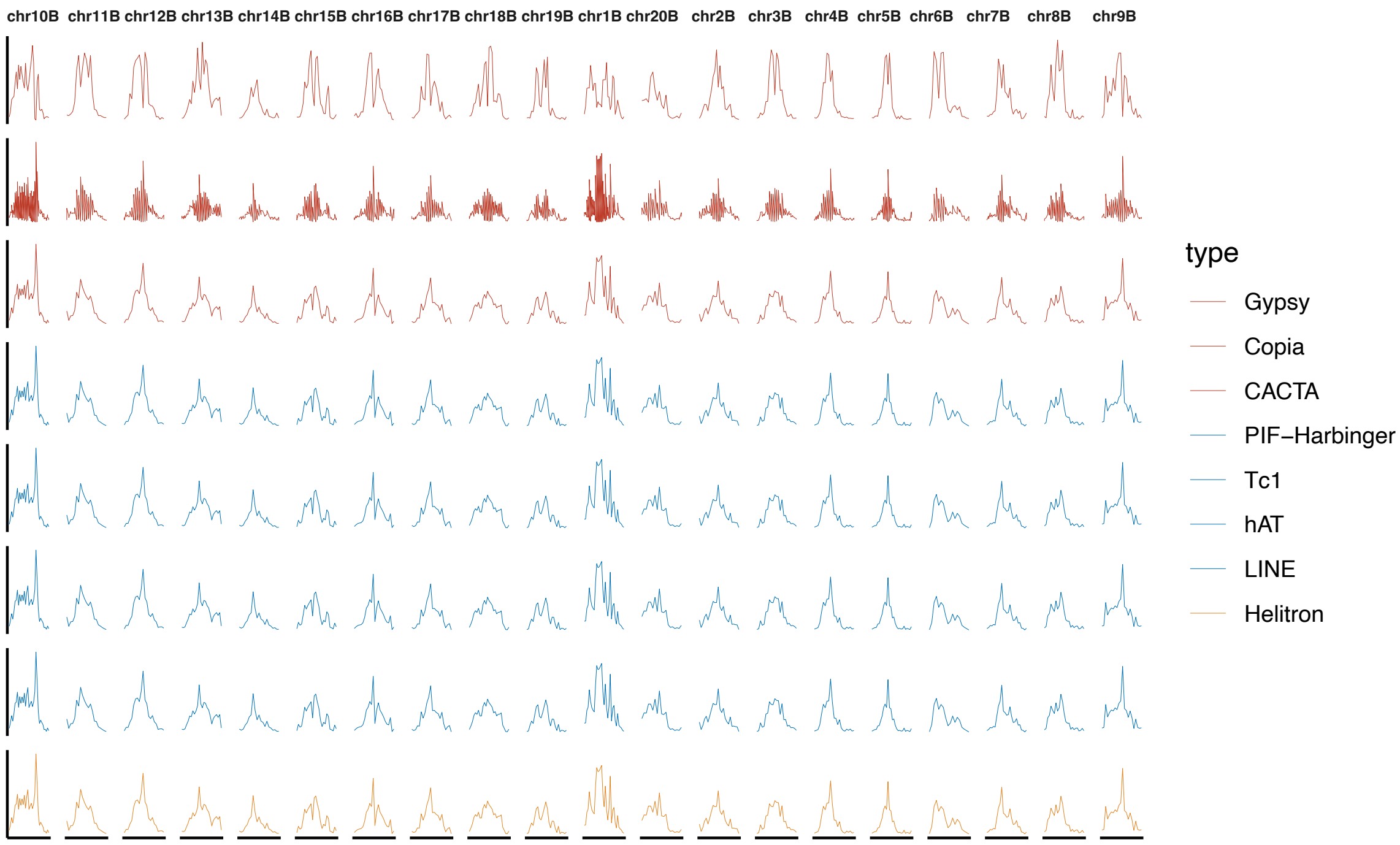
chr10B chr11B chr12B chr13B chr14B chr15B chr16B chr17B chr18B chr19B chr1B chr20B chr2B chr3B chr4B chr5B chr6B chr7B chr8B chr9B
type
Gypsy
Copia
CACTA
PIF–Harbinger
Tc1
hAT
LINE
Helitron

(**C**)

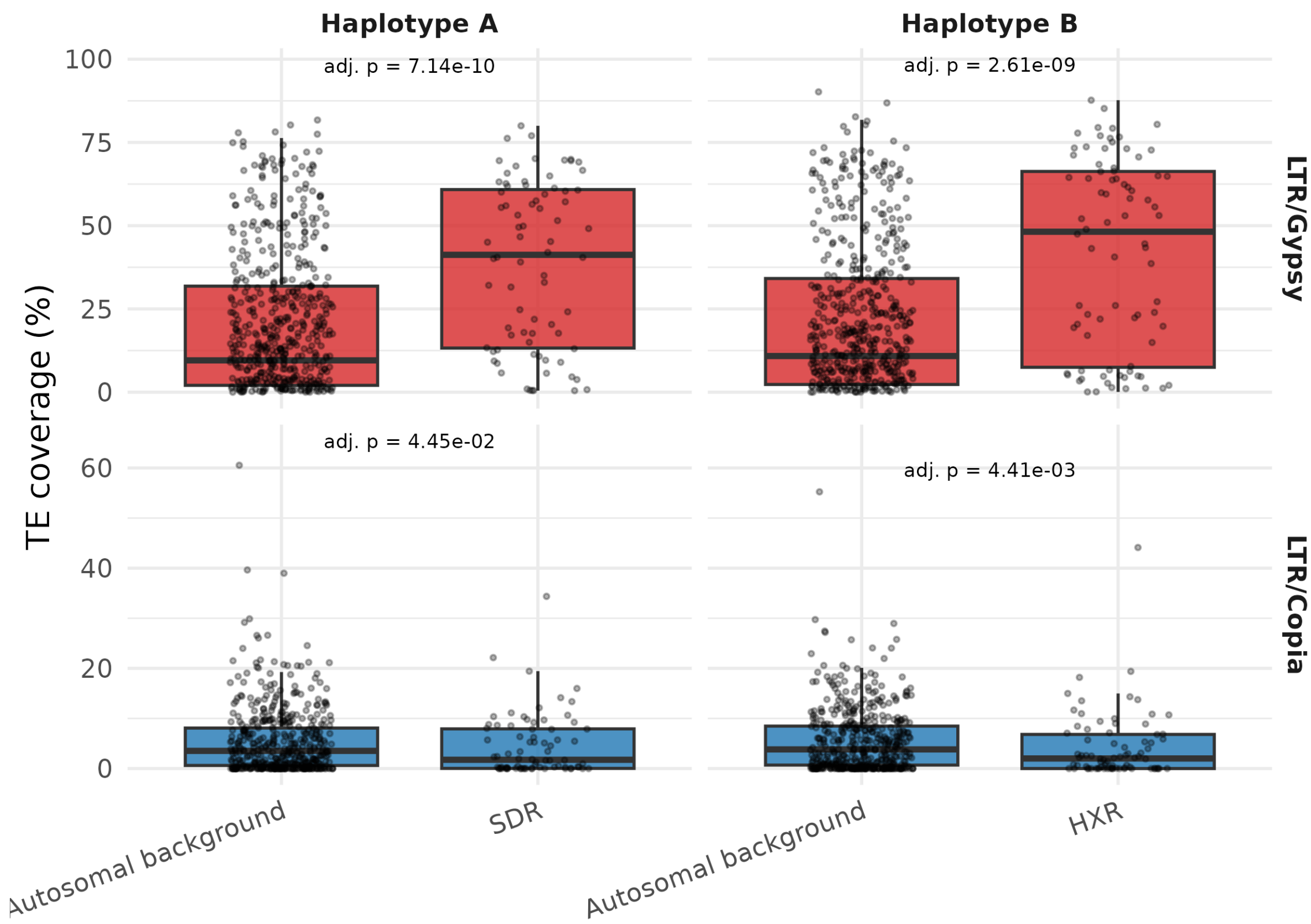
Haplotype A
Haplotype B
adj. p = 7.14e-10
adj. p = 2.61e-09
adj. p = 4.45e-02
adj. p = 4.41e-03
TE coverage (%)
100
75
50
25
0
60
40
20
0
LTR/Gypsy
LTR/Copia
Autosomal background
SDR
Autosomal background
HXR
