# Supplementary material for: Haplotype‐resolved genome assembly of a male Dioscorea alata cultivar reveals the structure and evolution of young sex chromosomes
Source: Plant J. 2026 Aug 17;127(4):e71091. doi: 10.1111/tpj.71091 (PMC13479938; doi:10.1111/tpj.71091)

Supplementary Figure 8: Collinearity analysis between the HaplotypeA, HaplotypeB of the 'Kabusa' genome and the female genome assembly from Bredeson et al. (2022).

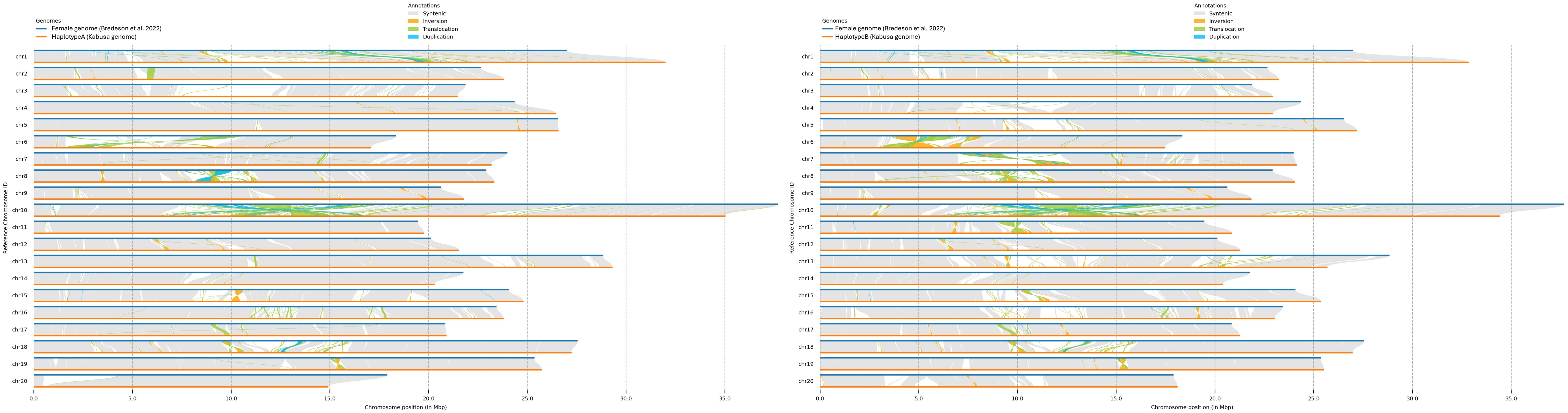

Supplement: Supplementary file 7 [file TPJ-127-0-s008.pdf]
