# Supplementary material for: Haplotype‐resolved genome assembly of a male Dioscorea alata cultivar reveals the structure and evolution of young sex chromosomes
Source: Plant J. 2026 Aug 17;127(4):e71091. doi: 10.1111/tpj.71091 (PMC13479938; doi:10.1111/tpj.71091)
Supplement: Supplementary file 8 [file TPJ-127-0-s009.pdf]

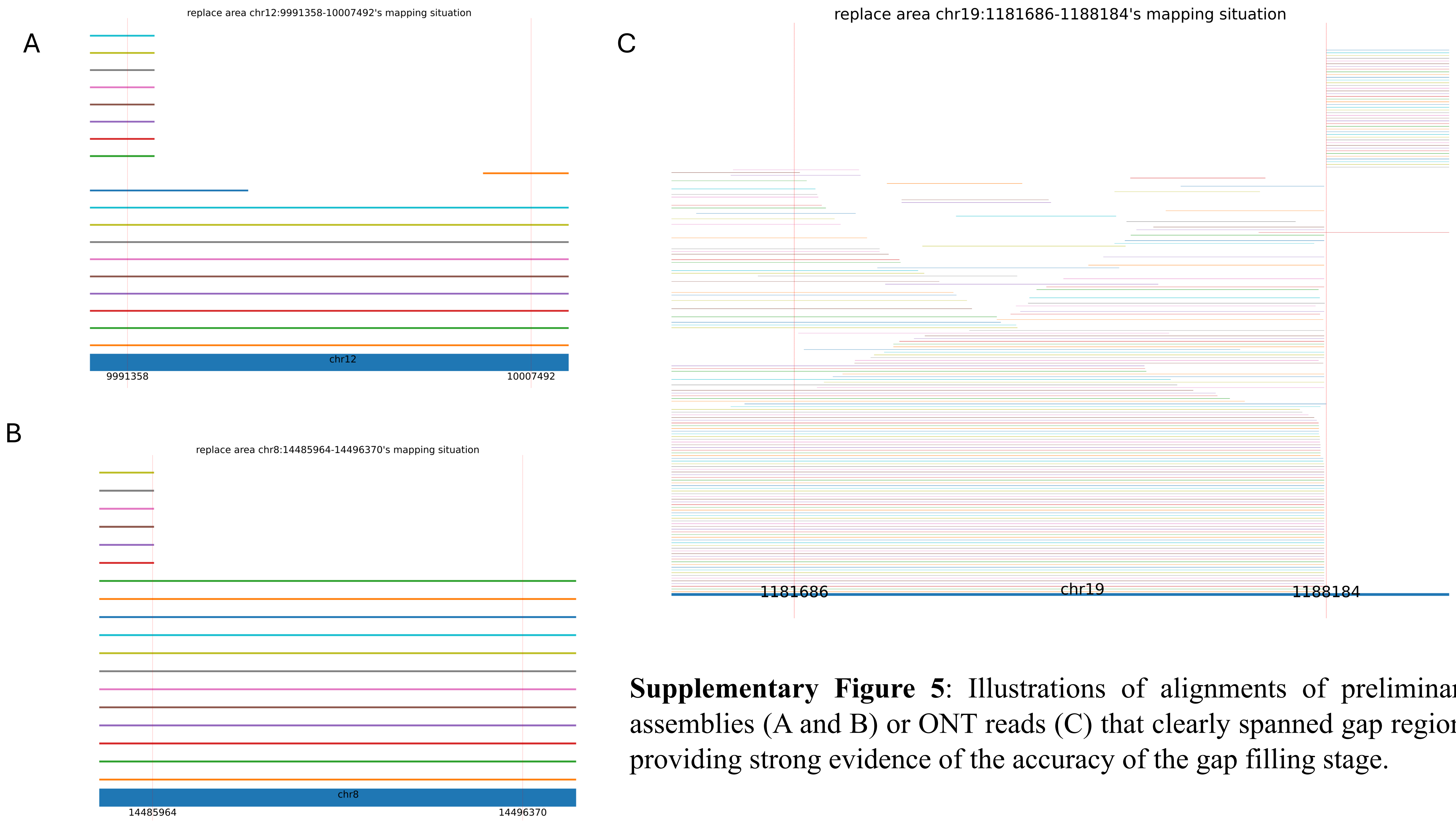

Supplementary Figure 5: Illustrations of alignments of preliminary assemblies (A and B) or ONT reads (C) that clearly spanned gap regions providing strong evidence of the accuracy of the gap filling stage.
