# Supplementary material for: Haplotype‐resolved genome assembly of a male Dioscorea alata cultivar reveals the structure and evolution of young sex chromosomes
Source: Plant J. 2026 Aug 17;127(4):e71091. doi: 10.1111/tpj.71091 (PMC13479938; doi:10.1111/tpj.71091)

**Supplementary Figure 16:** Genotyping of *D. alata* diversity panel (146 accessions from different countries) with the *sda6* PACE marker. Fluorescence signals are plotted by accession and observed sex. In x-axis, the "T" fluorescence allele (X), and in y-axis, the "G" fluorescence allele (Y).

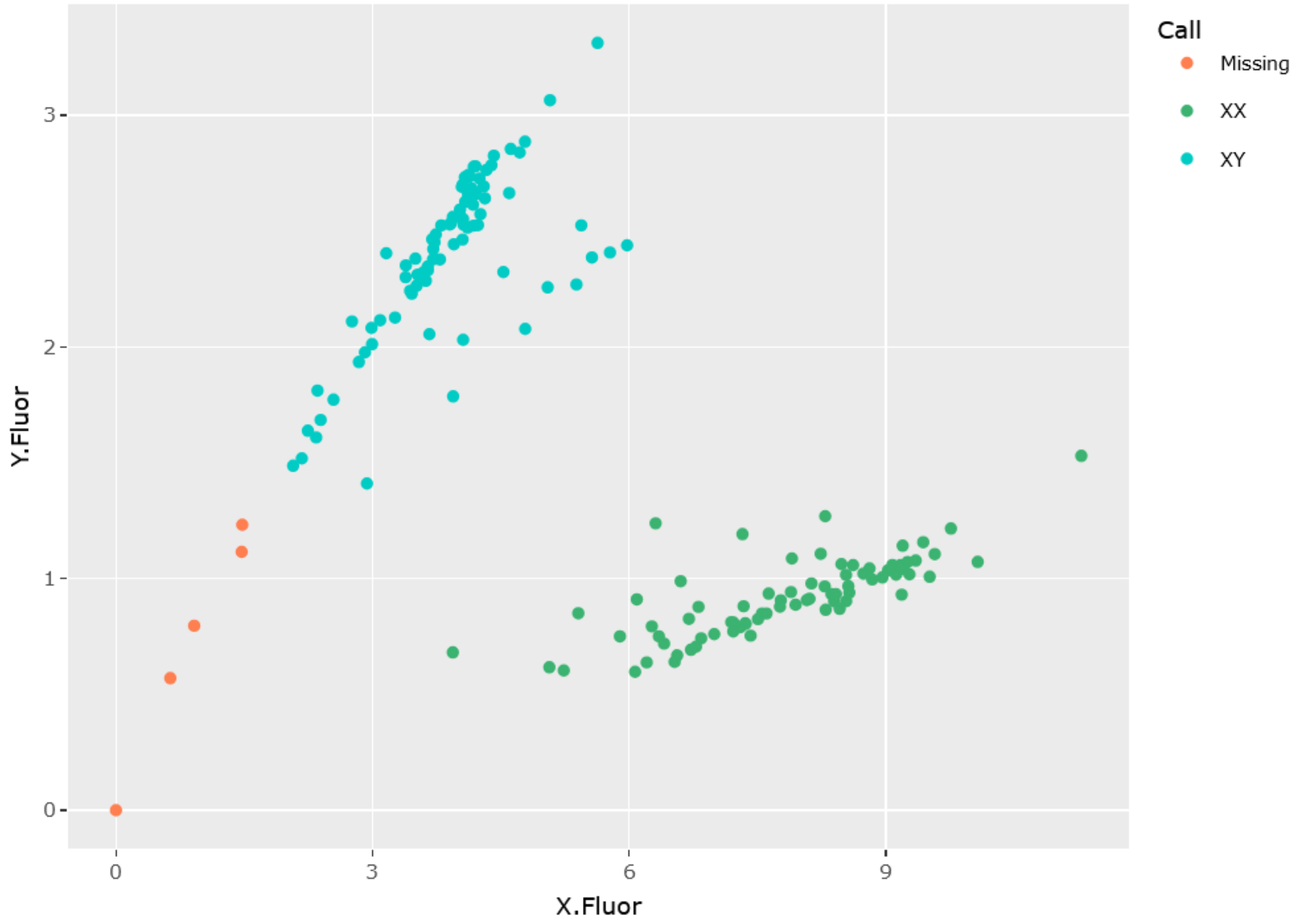

Supplement: Supplementary file 9 [file TPJ-127-0-s010.pdf]
