# Supplementary material for: Haplotype‐resolved genome assembly of a male Dioscorea alata cultivar reveals the structure and evolution of young sex chromosomes
Source: Plant J. 2026 Aug 17;127(4):e71091. doi: 10.1111/tpj.71091 (PMC13479938; doi:10.1111/tpj.71091)

Supplementary Figure 2: k-mer analysis for estimation of the 'Kabusa' genome size. The genome size was estimated to approximately 494.13 Mb.

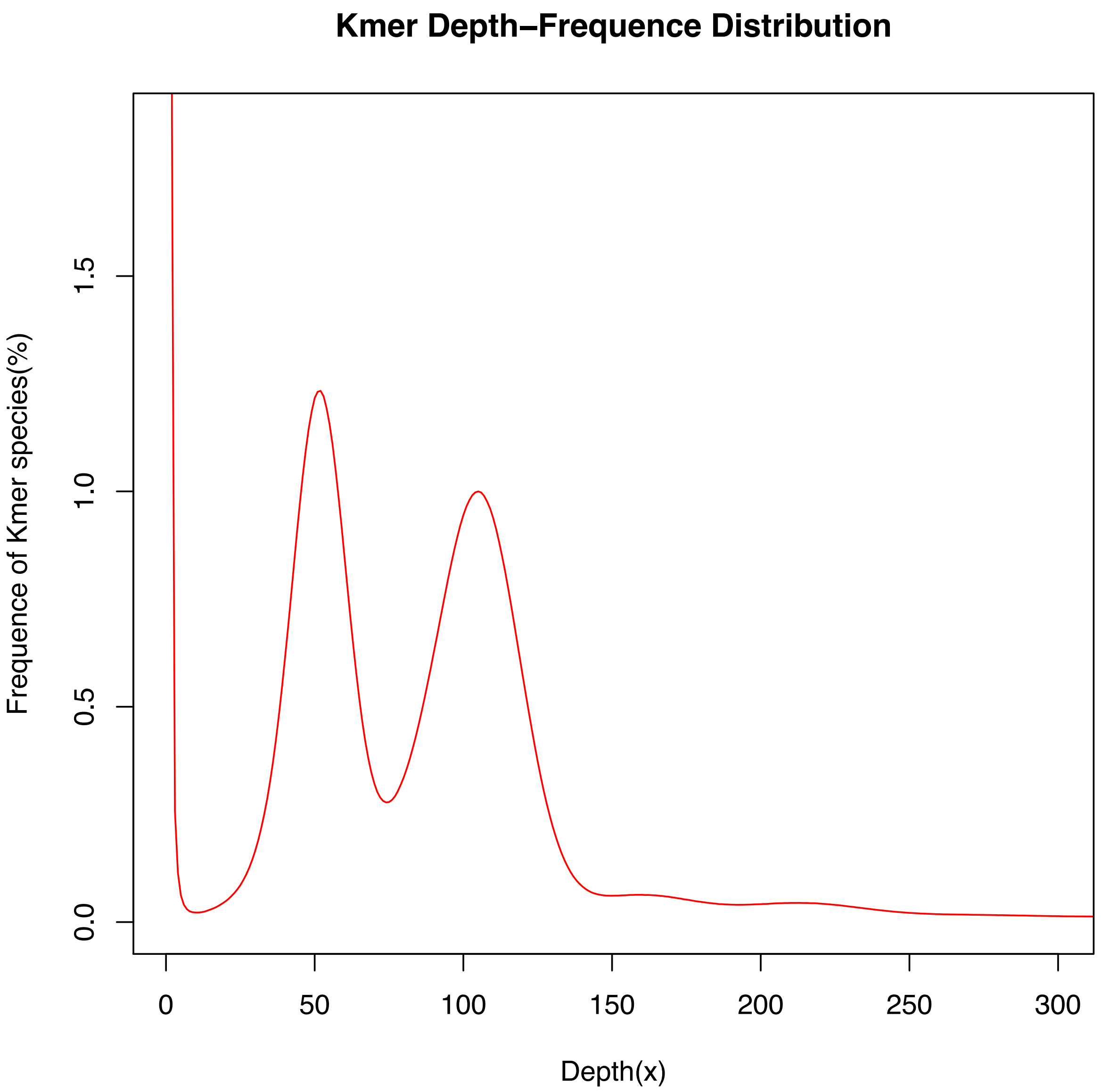

Supplement: Supplementary file 10 [file TPJ-127-0-s011.pdf]
