# Supplementary material for: Haplotype‐resolved genome assembly of a male Dioscorea alata cultivar reveals the structure and evolution of young sex chromosomes
Source: Plant J. 2026 Aug 17;127(4):e71091. doi: 10.1111/tpj.71091 (PMC13479938; doi:10.1111/tpj.71091)

Supplementary Figure 6: Centromere detection in the two haplotype genome assemblies of ‘Kabusa’. The first layer representing LTR/Copia density (blue color), the second layer representing LTR/Gypsy density (orange color), the third layer representing tandem repeat density (ruby red color) and the fourth layer representing the gene density (turquoise color) along the chromosomes.

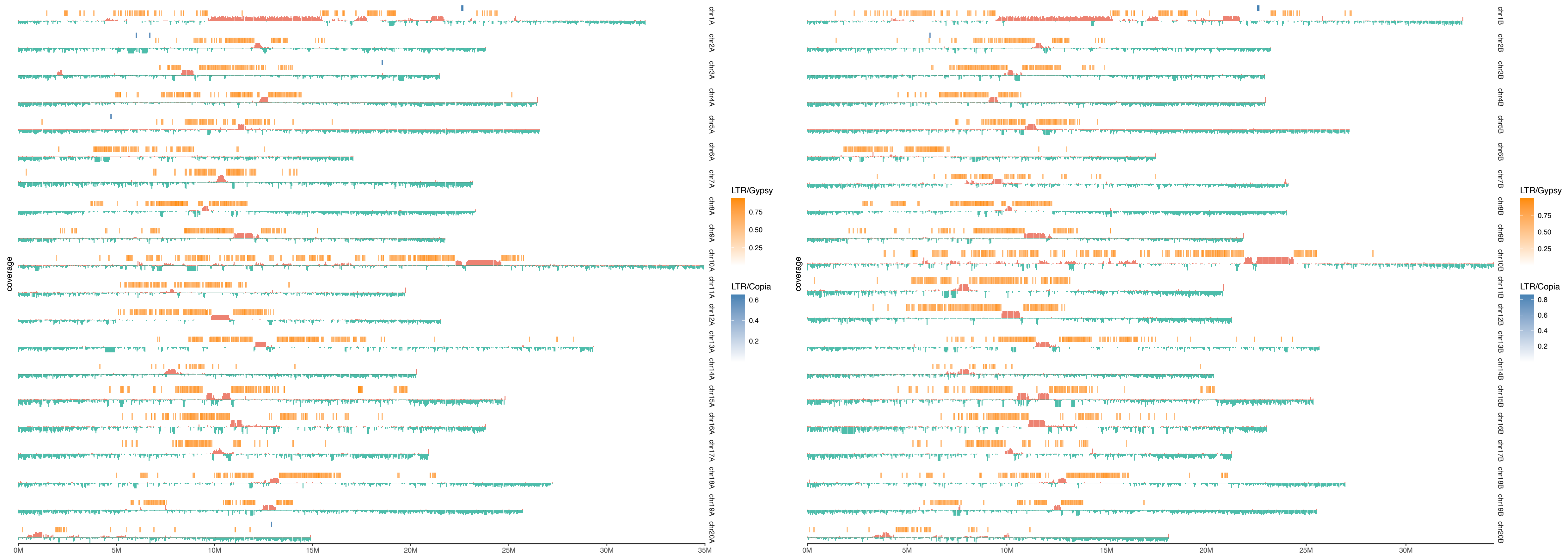

Supplement: Supplementary file 11 [file TPJ-127-0-s012.pdf]
