# Supplementary material for: Haplotype‐resolved genome assembly of a male Dioscorea alata cultivar reveals the structure and evolution of young sex chromosomes
Source: Plant J. 2026 Aug 17;127(4):e71091. doi: 10.1111/tpj.71091 (PMC13479938; doi:10.1111/tpj.71091)

Supplementary Figure 15: Patterns of methylation level (DNA methylation 5mC at CpG sites) along homologous chromosomes in the two haplotype assemblies of the ‘Kabusa’ genome. Plot was generated using a window of 100 kb.

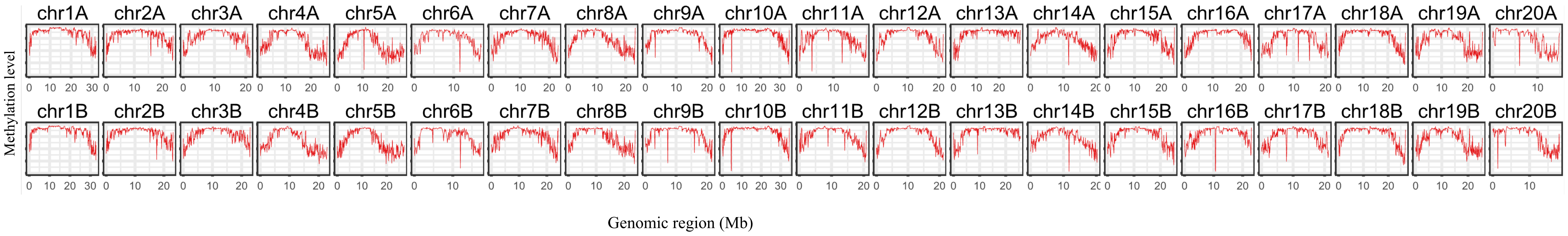

Supplement: Supplementary file 12 [file TPJ-127-0-s013.pdf]
