# Supplementary material for: Haplotype‐resolved genome assembly of a male Dioscorea alata cultivar reveals the structure and evolution of young sex chromosomes
Source: Plant J. 2026 Aug 17;127(4):e71091. doi: 10.1111/tpj.71091 (PMC13479938; doi:10.1111/tpj.71091)

Supplementary Figure 1: Flow cytometry-based determination of the ploidy level in 'Kabusa'. The ploidy level was assessed by comparing the position of the main peak with the references indicated by the arrows. According to the results, 'Kabusa' is identified as a diploid cultivar (2X).

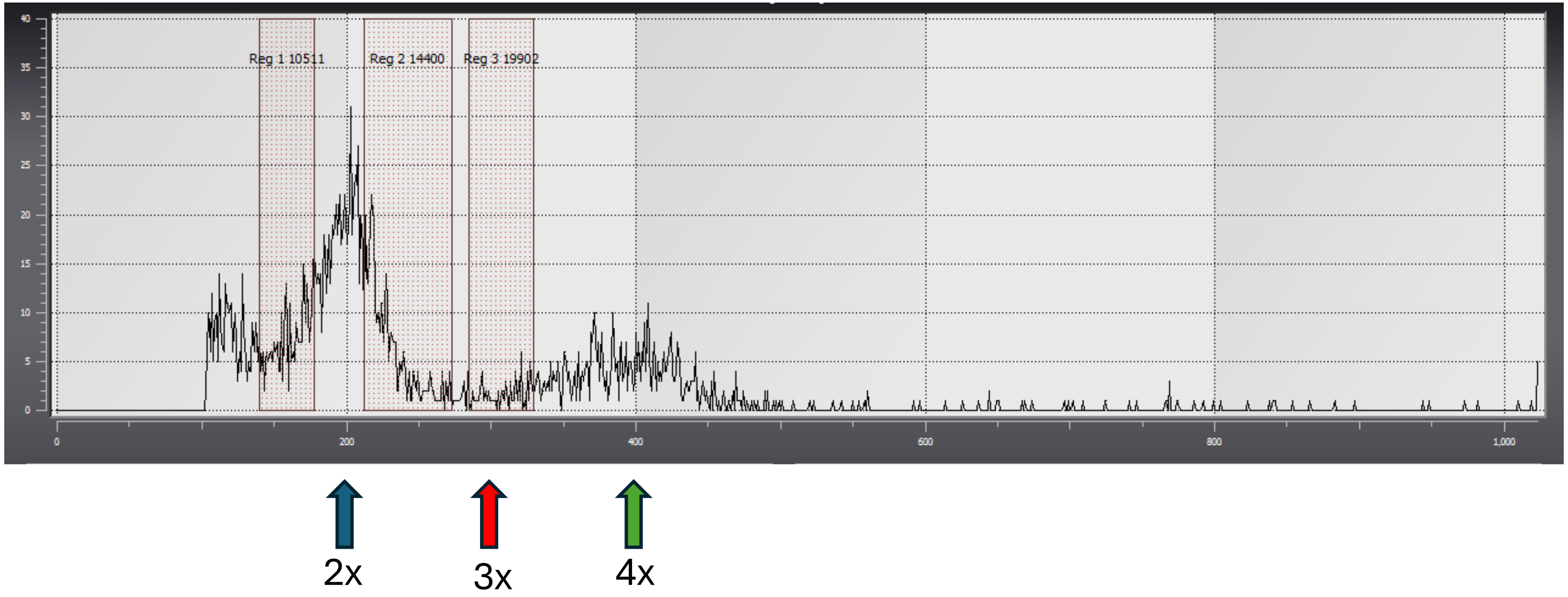

Supplement: Supplementary file 13 [file TPJ-127-0-s014.pdf]
