# Supplementary material for: Haplotype‐resolved genome assembly of a male Dioscorea alata cultivar reveals the structure and evolution of young sex chromosomes
Source: Plant J. 2026 Aug 17;127(4):e71091. doi: 10.1111/tpj.71091 (PMC13479938; doi:10.1111/tpj.71091)

Supplementary Figure 11 : Sex chromosome evolution in yams. (A) Synteny analysis between genomes of four yam species with emphasis on sex chromosomes reported to date. Dala_V2 = female genome of *Dioscorea alata*, DalA and DalB = Two haplotype assemblies of the 'Kabusa' genome (*D. alata*), Drot = *D. rotundata*, Dtok = *D. tokoro* and Dzin = *D. zingiberensis*. *Dioscorea alata* and *D. rotundata* belong to the section Enantiophyllum while *D. tokoro* and *D. zingiberensis* belong to the section Stenophora. XY and ZW denote the sex determination systems reported in the yam species. Numbers before and within brackets represent divergence time million years ago

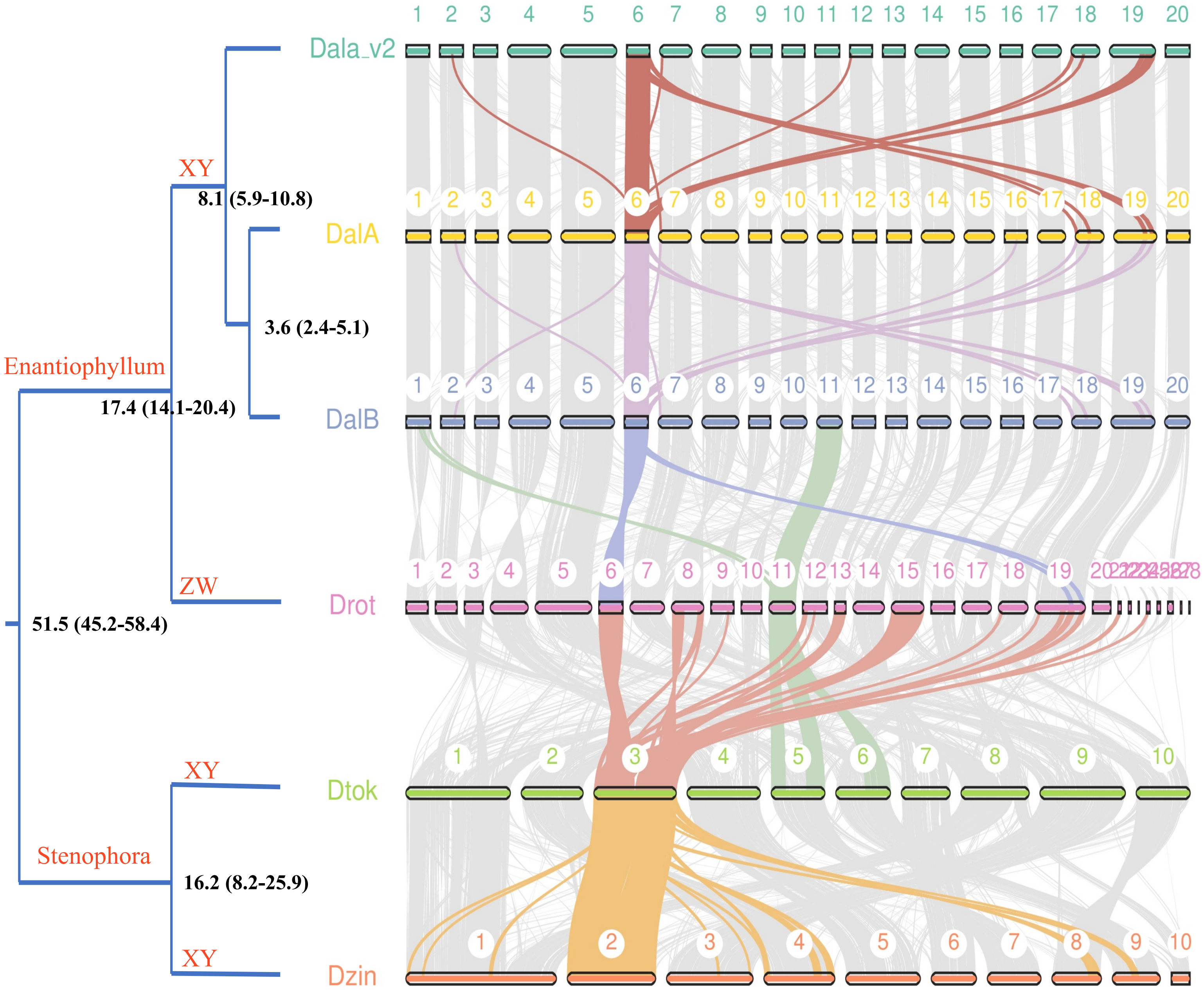

Supplement: Supplementary file 14 [file TPJ-127-0-s015.pdf]
