# Supplementary material for: Haplotype‐resolved genome assembly of a male Dioscorea alata cultivar reveals the structure and evolution of young sex chromosomes
Source: Plant J. 2026 Aug 17;127(4):e71091. doi: 10.1111/tpj.71091 (PMC13479938; doi:10.1111/tpj.71091)

Supplementary Figure 4: Centromere and telomere detection map in the two haplotype genome assemblies of 'Kabusa'. Orange squares and green triangles represent centromere and telomeres within the assembled genome. Blue indicates low gene density; red indicates high gene density. Deep blue and light blue circles represent the distribution along the chromosomes of 45S_rDNA and 5S_rDNA, respectively.

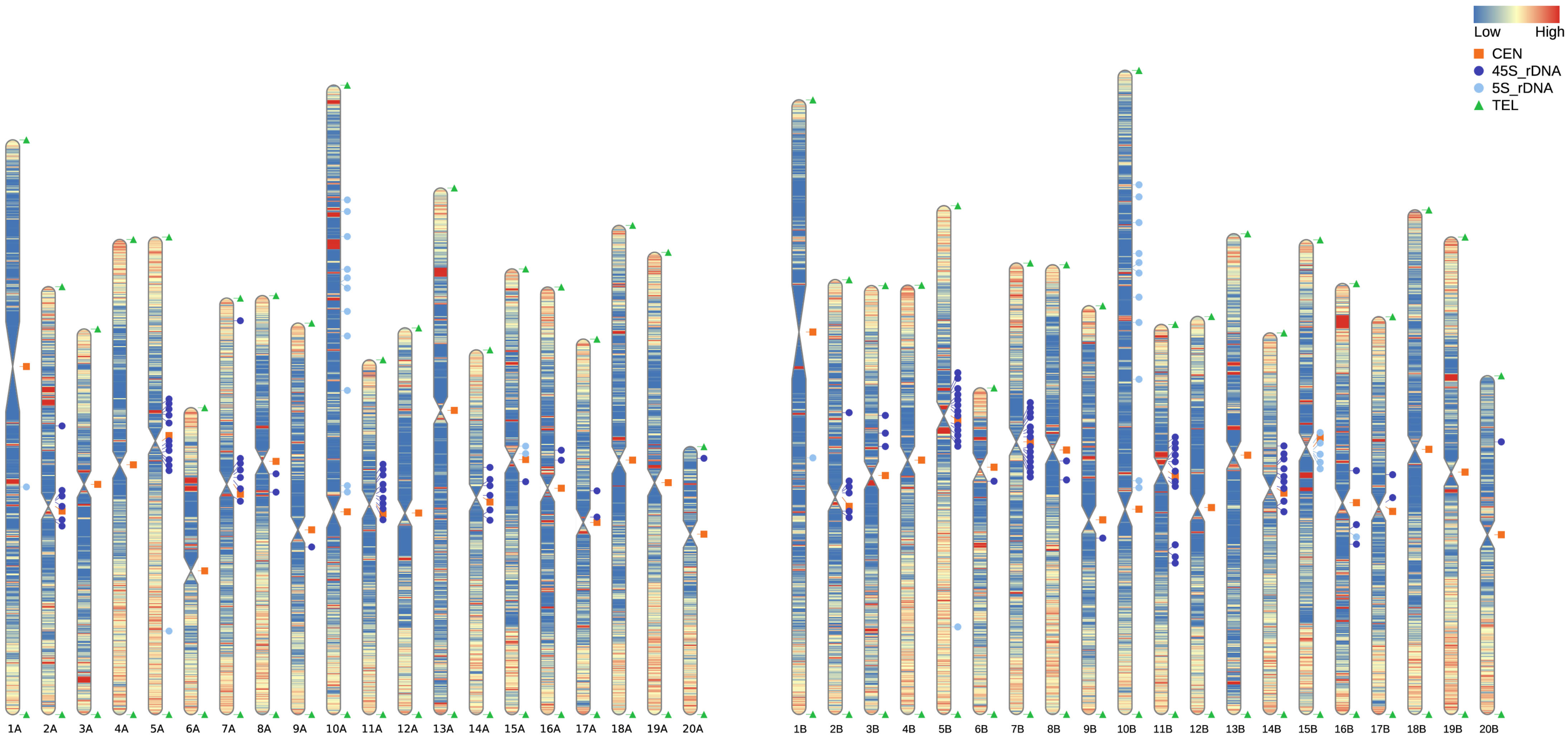

Supplement: Supplementary file 15 [file TPJ-127-0-s016.pdf]
