# Supplementary material for: Haplotype‐resolved genome assembly of a male Dioscorea alata cultivar reveals the structure and evolution of young sex chromosomes
Source: Plant J. 2026 Aug 17;127(4):e71091. doi: 10.1111/tpj.71091 (PMC13479938; doi:10.1111/tpj.71091)

Supplementary Figure 9: Intergenomic comparison of the HaplotypeA, HaplotypeB of the 'Kabusa' genome and the female genome assembly from Bredeson et al. (2022). Red circle shows the location of the pericentric inversion in DalaChr6A vs DalaChr6F but absent in DalaChr6B vs DalaChr6F.

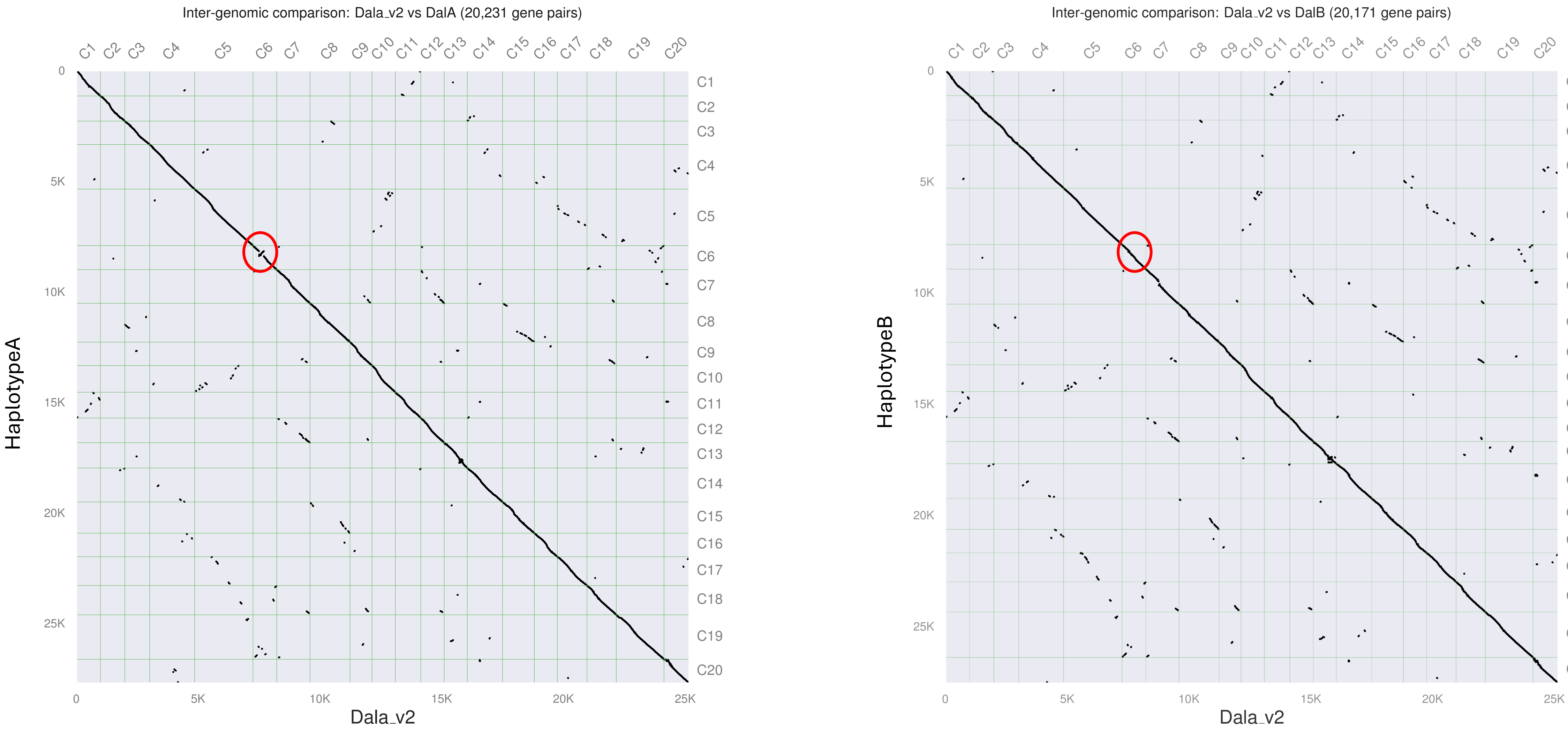

Supplement: Supplementary file 16 [file TPJ-127-0-s017.pdf]
